# Nucleoside transporter-guided cytarabine-conjugated liposomes for intracellular methotrexate delivery and cooperative choriocarcinoma therapy

**DOI:** 10.1186/s12951-021-00931-3

**Published:** 2021-06-15

**Authors:** Weidong Fei, Yunchun Zhao, Xiaodong Wu, Dongli Sun, Yao Yao, Fengmei Wang, Meng Zhang, Chaoqun Li, Jiale Qin, Caihong Zheng

**Affiliations:** 1 Department of Pharmacy, Women’s Hospital, Zhejiang University School of Medicine, Hangzhou, 310006 China; 2 Department of Gynecologic Oncology, Women’s Hospital, Zhejiang University School of Medicine, Hangzhou, 310006 China; 3 Department of Ultrasound, Women’s Hospital, Zhejiang University School of Medicine, Hangzhou, 310006 China

**Keywords:** Equilibrative nucleoside transporter 1, Choriocarcinoma, Methotrexate, Cytarabine, Cooperative therapy

## Abstract

Gestational trophoblastic tumors seriously endanger child productive needs and the health of women in childbearing age. Nanodrug-based therapy mediated by transporters provides a novel strategy for the treatment of trophoblastic tumors. Focusing on the overexpression of human equilibrative nucleoside transporter 1 (ENT1) on the membrane of choriocarcinoma cells (JEG-3), cytarabine (Cy, a substrate of ENT1)-grafted liposomes (Cy-Lipo) were introduced for the targeted delivery of methotrexate (Cy-Lipo@MTX) for choriocarcinoma therapy in this study. ENT1 has a high affinity for Cy-Lipo and can mediate the endocytosis of the designed nanovehicles into JEG-3 cells. The ENT1 protein maintains its transportation function through circulation and regeneration during endocytosis. Therefore, Cy-Lipo-based formulations showed high tumor accumulation and retention in biodistribution studies. More importantly, the designed DSPE-PEG_2k_-Cy conjugation exhibited a synergistic therapeutic effect on choriocarcinoma. Finally, Cy-Lipo@MTX exerted an extremely powerful anti-choriocarcinoma effect with fewer side effects. This study suggests that the overexpressed ENT1 on choriocarcinoma cells holds great potential as a high-efficiency target for the rational design of active targeting nanotherapeutics.

**Supplementary Information:**

The online version contains supplementary material available at 10.1186/s12951-021-00931-3.

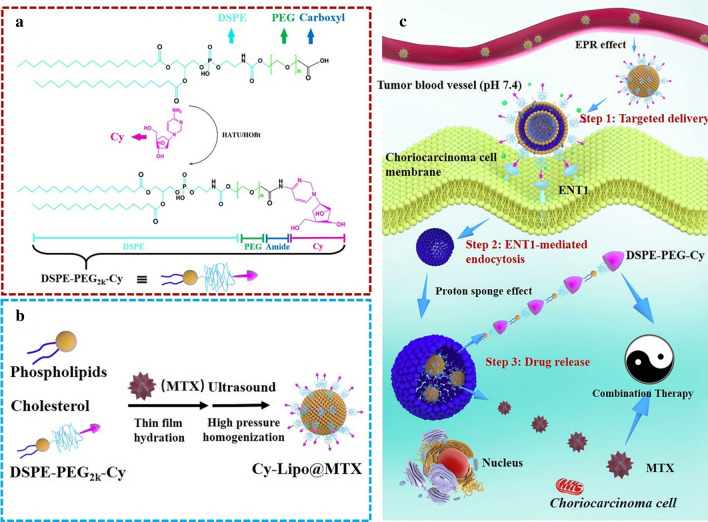

## Background

Gestational trophoblastic neoplasia (GTN) is a condition wherein placental trophoblasts proliferate excessively, giving rise to malignancies such as placental site trophoblastic tumors, invasive moles, choriocarcinoma, and epithelioid trophoblastic tumors [1, 2]. Half of all GTN cases arise from a molar pregnancy, with the remainder following an ectopic pregnancy, term pregnancy, or spontaneous abortion [3]. With the implementation of the “two-child” policy in China, women's reproductive needs have increased, and the incidence of GTN has also increased significantly. Among GTNs, choriocarcinoma is a highly aggressive gestational trophoblastic condition affecting 1/50,000 pregnancies (Additional file 1: Fig. S1a) [4]. Choriocarcinoma leads to extensive local trophoblast invasion and vascular permeation that can result in renal, hepatic, pulmonary, and brain metastasis [5, 6]. Without the early diagnosis and appropriate treatment, choriocarcinoma may cause severe bleeding, uterine rupture, hysterectomy, etc., all of which seriously threaten the reproductive health and lives of women; therefore, it has attracted great attention in the clinic. Among the clinical chemotherapeutic strategies, methotrexate (MTX) is the most widely used first-line drug [7]. However, the poor specificity of MTX is likely to cause adverse reactions such as liver damage, abdominal pain, gastrointestinal reactions, mucosal damage, and bone marrow suppression during systemic administration (incidence rate of more than 30%) [8]. More seriously, some of these cases will result in serious adverse reactions such as allergic pneumonia and convulsions [9]. The above treatment status causes serious damage to the normal tissues of patients in childbearing age.

With the development of nanotechnology and nanoscience, great progress has been made in drug delivery to specific lesions and for cancer therapy [10, 11]. Targeted nanodrugs that can recognize specific markers on the tumor surface and enrich at the tumor sites based on the principle of ligand/antibody binding to cell membrane receptors/antigens have achieved better treatment effects than non-targeted nanodrugs [12, 13]. However, although research in this field has been carried out for decades, few active targeting nanodrugs have been approved for use in the clinic [14]. One of the biggest issues is the unpredictable targeting efficacy, which is oftentimes not uniform between individuals due to the high variability and heterogeneity in the expression of receptors [15]. Another fundamental reason is that macromolecular ligands (such as EGFR, LDL, transferrin, angiopep, etc.) have strong immunogenicity and steric hindrance, resulting in fast clearance from the body and low targeting efficiency [16–18]. Therefore, the construction of more efficient targeted drug delivery strategies is important for trophoblastic tumor therapy.

Membrane transporters, such as glucose transporters, amino acid transporters, choline transporters, and nucleoside transporters, are essential to mammalian cell nutrition, providing cells with glucose, amino acids, vitamins, pyrimidines, ions, and other vital nutrients [19]. Furthermore, transporters are also involved in the transport of various therapeutic drugs. For example, human equilibrative nucleoside transporter 1 (ENT1) can efficiently transport gemcitabine [20], cytarabine (Cy), and other drugs into the cell [21, 22]. As a result, transporters act as the key factors that determine the pharmacological effects and safety of drugs [23, 24]. The latest research has found that nanodrugs conjugated with specific membrane transporter substrates can not only achieve targeted drug delivery but can also enter cancer cells through mediation by the transporters. For instance, the research group of Jiang C designed choline transporter-mediated nanoplatforms to treat glioma [25, 26]. These nanodrugs exhibited improved therapeutic activity when modified with choline derivates. The research group of Sun J demonstrated that the Na^+^-coupled transporter OCTN2 or amino acid transporters ATB^0,+^ and LAT1 overexpressed on the membrane of cancer cells could transport small molecule substrate-modified nanodrugs into tumor cells [27–29]. These nanodrugs exerted superior antitumor effects compared to that of unmodified formulations. All of the above studies indicated that the highly expressed transporter proteins on tumor cell membranes could be new promising targets for specific drug delivery.

Rapid proliferation is a characteristic of tumor tissues. In addition to their high uptake capacity of amino acids and biotin, the cancer cell demand for nucleosides is also much greater than that of normal cells. Nucleosides require specific transporters to penetrate the cell membrane. Among the family of nucleoside transporters, ENT1 is the most widely expressed and highly selective transporter of purines and pyrimidine nucleosides [30]. Some studies have reported that ENT1 could be used as a bona fide target for potential drugs related to cancer therapy [31–34]. Our group found that ENT1 is overexpressed on choriocarcinoma cells (JEG-3 cells) by analyzing the expression of transporters. Inspired by this result, it was hypothesized that ENT1 may act as a novel and effective tumor target for nanotherapeutics. Cytarabine (Cy), which can be specifically transported into cells via ENT1 as an analogue of the nucleoside cytosine [22, 35, 36], was selected as a guide for targeted choriocarcinoma therapy. Furthermore, Cy is a cell cycle-specific drug that can interfere with the metabolism of nucleic acids and has therapeutic effects on human choriocarcinoma cells [37, 38].

**Fig. 1:**
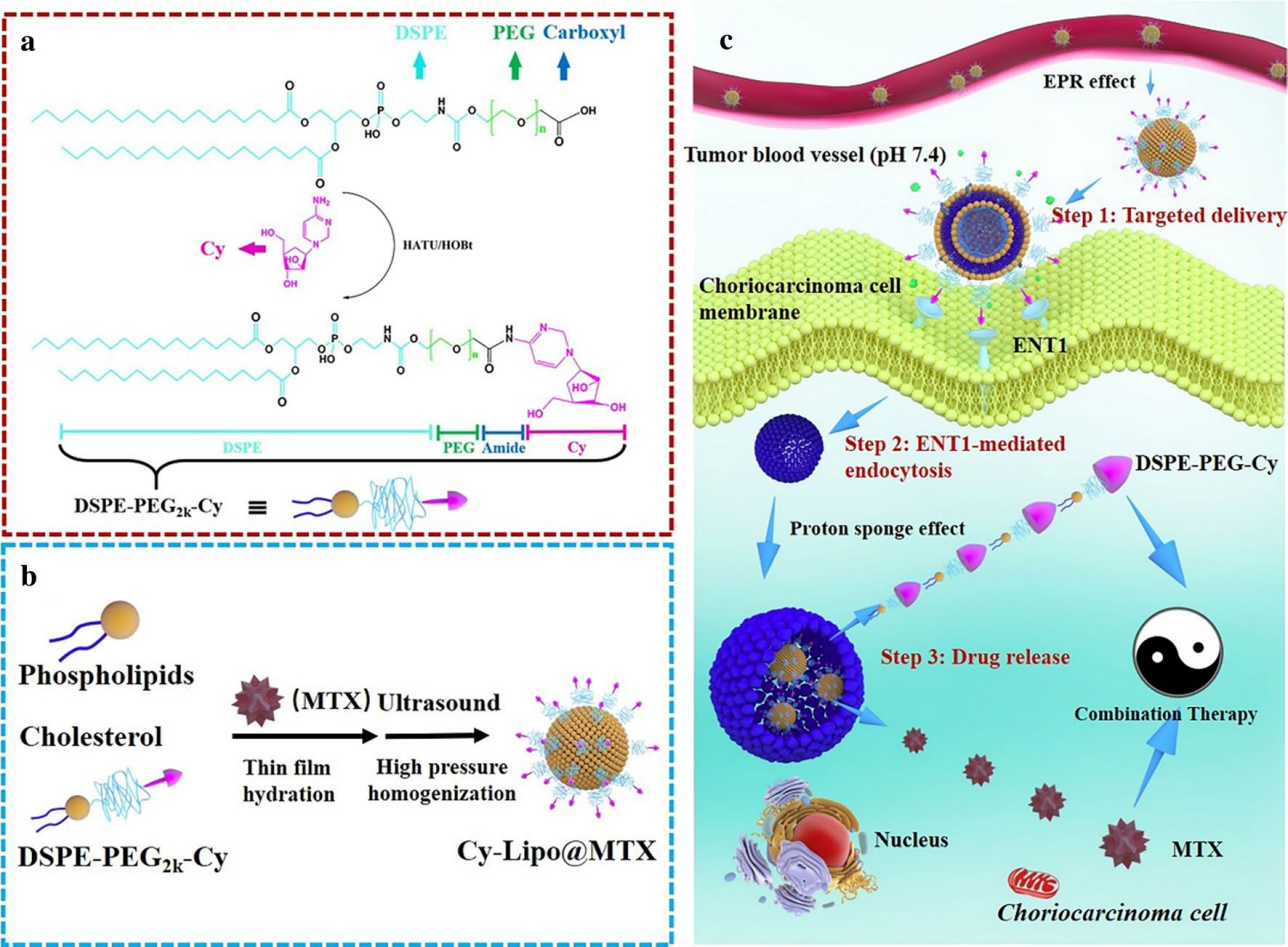
Synthesis diagram of DSPE-PEG_2k_-Cy (**a**). Preparation process of Cy-Lipo@MTX (**b**). Schematic illustration of Cy-Lipo@MTX for choriocarcinoma therapy (**c**)

In this research, Cy-conjugated distearoylphosphatidylethanolamine-polyethylene glycol (DSPE-PEG_2k_-Cy, abbreviated as Cy-lipid) was synthesized through a condensation reaction between the amine of Cy and the active carbonyl group of carboxyl-terminated PEG-lipids (Fig. 1a). Then, MTX-loaded liposomes (Cy-Lipo@MTX) were prepared by the thin film hydration method combined with the high-pressure homogenization method (Fig. 1b). The morphology, stability, release pattern, cellular uptake, and cytotoxicity of the nanodrugs were characterized. Then, the pharmacokinetic and distribution features of Cy-Lipo@MTX were investigated in mice. More importantly, the mechanism of ENT1-mediated endocytosis and the therapeutic effects of the nanodrugs were explored (as shown in Fig. 1c). This study comprehensively evaluates the role of ENT1 in the treatment of choriocarcinoma and provides a novel concept to design transporter-guided targeted drug delivery for cancer therapy.

## Experimental section

### Materials

Methotrexate hydrate, gemcitabine, cytosine β-d-arabinofuranoside, doxorubicin, and 2′-deoxycytidine hydrochloride were obtained from Aladdin Bio-Chem Technology Co., Ltd (Shanghai, China). DSPE-PEG_2k_-COOH was supplied by Ruixi Biological Technology Co., Ltd (Xi’an, China). Soybean phospholipids and cholesterol were purchased from A.V.T Pharmaceutical Co., Ltd (Shanghai, China). Dialysis tubes (MW: 3500 D) were purchased from Spectrum Laboratories, Inc. (CA, USA). Dimethyl sulfoxide (DMSO), IR-780 iodide, and MTT were obtained from Sigma Aldrich (MO, USA). DMEM, RPMI-1640, fetal bovine serum (FBS), penicillin G sodium, and streptomycin sulfate were obtained from Gibco BRL (MD, USA). The FITC Annexin V Apoptosis Detection Kit I and the PI/RNase Staining kit were purchased from BD Biosciences (CA, USA). MitoProbe™ JC-1 Assay kit and DAPI were purchased from Thermo Fisher Scientific (MA, USA). Protein extraction kit was obtained from Beyotime Biotechnology Co., Ltd. (Shanghai, China). All other compounds were analytical grade, and a Millipore system was used to purify water.

### Cell culture and animals

JEG-3 (choriocarcinoma cells) and HepG2 (human liver cancer cells) were cultured in Eagle's Minimum Essential Medium or DMEM containing 10% FBS and penicillin/streptomycin (100 U/mL), respectively. Pricell-0051 (normal human placental trophoblast cells) and MCF-7 (human breast cancer cells) cell lines were cultured in DMEM/F12 or RPMI-1640 containing 10% FBS and penicillin/streptomycin (100 U/mL) in a 37 °C 5% CO_2_ incubator. Female Sprague–Dawley (SD) rats (220 ± 20 g) and female nude BALB/c mice (20 ± 2 g) were obtained from the Laboratory Animal Center of Zhejiang Chinese Medical University.

### High expression of ENT1 in JEG-3 cells

Firstly, the mRNA expression of various kinds of cell membrane transporters was evaluated in JEG-3 cells, including human equilibrative nucleoside transporters (ENT), concentrative nucleoside transporters (CNT), organic anion transporters (OATs), organic cation transporters (OCTs), carnitine/organic cation transporters (OCTNs), multidrug resistance-associated protein (MRPs), P-glycoprotein (P-gp), and breast cancer resistance protein (BCRPs). JEG-3 cells were grown to about 80% confluence in a 10 cm dish and collected for RT-PCR analysis. Total RNA was extracted from the cultured JEG-3 cells using TRIzol reagent (Ambion, Thermo Fisher Scientific, USA). High-Capacity cDNA Reverse Transcription kit (Applied Biosystems, Carlsbad, USA) was employed to prepare cDNA from RNA (2 µg), and cDNA was analyzed via RT-PCR with SYBR Premix Ex Taq (Takara, Dalian, China). The PCR primers of transporters were listed in Additional file 1: Table S1.

For western blot analysis, JEG-3 cells, MCF-7 cells, or HepG2 cells were plated overnight in 6-well plates. The cell lysates were collected and assessed via 13% denaturing polyacrylamide gel electrophoresis. An ultrasonic cell disruptor was used to extract proteins from cells, after which a Bio-Rad Electrophoresis instrument was used for quantification. Proteins were then separated via SDS-PAGE, transferred to PVDF membranes, and incubated with primary antibodies of ENT1 overnight. Secondary antibodies were then used to probe blots. A gel imaging system was then used for protein detection.

### Specific uptake of cytarabine into the cells

JEG-3 cells, MCF-7 cells, or HepG2 cells were plated overnight in 6-well plates. Then, the adhered cells were treated with Cy (5 μM) for 30 min or 60 min. Then, cells were washed with PBS for moving the free drug in the medium. After that, the cells were lysed and treated with acetonitrile for precipitating protein. Finally, the content of Cy was detected by LC/MS (8050, SHIMADZU, Japan).

### Synthesis and characterization of DSPE-PEG2k-Cy

Firstly, 140.00 mg of DSPE-PEG_2k_-COOH was dissolved in dimethylformamide. Then, cytarabine, 2-(7-Azabenzotriazol-1-yl)-N, N, N', N'-tetramethyluronium hexafluorophosphate (HATU), triethylamine, and 1-hydroxybenzotriazole (HOBt) were added to the dimethylformamide system. The mixture was stirred for 3–5 h in an ice bath. The molar ratio of DSPE-PEG_2k_-COOH, cytarabine, HATU, HOBt, and triethylamine was 1:1.2:1.2:1.2:2. After that, the reaction solution was moved to a dialysis bag (MWCO = 3500 Da), and dialyzed in 1000 mL of deionized water for 72 h. Finally, DSPE-PEG_2k_-Cy was freeze-dried for further usage. The molecular weight and structure of DSPE-PEG_2k_-Cy were analyzed by the MALDI-TOF–MS (GCT-Premier, Waters, USA) and the Nicolet 6700 FT-IR spectrophotometer (Thermo Electron Corporation, USA).

### Preparation and characterization of Lipo@MTX and Cy-Lipo@MTX

The MTX powder was dissolved in 0.5 M NaOH solution. Then the pH of the solution was adjusted to 7.0–8.0 with HCl solution. The uniform and stable MTX solution obtained through the above method was used for preparing the following MTX formulations. Cy-Lipo@MTX was prepared by the thin film hydration method combined with the high-pressure homogenization method. In brief, soybean phospholipids, cholesterol, and DSPE-PEG_2k_-Cy (mass ratio: 4.5:1.0:1.0) were added to a round-bottom flask and dissolved with chloroform. Then, a yellow film was formed by rotating on a rotary evaporator for 2 h. Then, the round-bottom flask was placed in a vacuum drying oven overnight to remove the residual chloroform. After that, MTX solution was added to the round-bottom flask with hydration for about 30 min at 40 °C. During this process, the clear yellow solution gradually turned into a yellow colloidal solution. Then, the hydrated liquid was sonicated for 3–5 min and homogenized 3 times with a high-pressure homogenizer (AH110D, ATS Engineering Inc., Canada). The redundant free drugs were removed by an ultrafiltration system (Labscale TFF system P6DA7080001, Millipore, USA). Finally, the dispersion was freeze-dried (FreeZone 2.5, LABCONCO, USA) with 5% (w/v) mannitol as cryoprotectant. The preparation of Lipo@MTX was to replace DSPE-PEG_2k_-Cy with an equivalent weight of DSPE-PEG_2k_-COOH, and the other preparation methods were described above. The preparation of fluorescein (doxorubicin (Dox) or IR780)-labeled liposomes was to replace MTX with fluorescein solution, and the other preparation methods were described above. Transmission electron microscopy images (TEM) of Lipo@MTX and Cy-Lipo@MTX were obtained by a transmission electron microscope (H-7650, Hitachi, Japan). The differential scanning calorimetry (DSC) curves of samples were scanned from 0 to 200 °C at a heating rate of 10 °C/min using a differential scanning calorimeter (DSC 200F3, NETZSCH, Germany).

### Drug loading efficiency and in vitro drug release

To measure the amount of MTX encapsulated within liposomes, the lyophilized Cy-Lipo@MTX or Lipo@MTX were dissolved in acetonitrile solution. The obtained solution was filtered through a 0.22 µm filter membrane. Then, high-performance liquid chromatography (HPLC) (1100, Agilent, USA) was used to determine drug concentrations [39]. The drug loading rate (DL%) and entrapment efficiency (EE%) were assessed as follows:

$${\text{DL}}\% \, = \,\left( {{\text{amount of MTX in the sample}}/{\text{total weight of formulations}}} \right)\, \times \,100\%$$

$${\text{EE}}\% \, = \,\left( {{\text{amount of MTX in the sample}}/{\text{total amount of MTX added in preparation}}} \right)\, \times \,{\text{1}}00\%$$

In vitro release profiles of MTX-loaded liposomes were determined at 37 °C in PBS or acetate buffer with pH of 7.4 or 5.5. Typically, 5 mL of Cy-Lipo@MTX or Lipo@MTX were added to a dialysis tube (MWCO 3500 Da). Next, the dialysis tube was placed in 250 mL of buffer and constantly stirred (100 rpm) at 37 °C. At specific time points, 2 mL of the external buffer was extracted and an equal amount of fresh medium was added. HPLC was used to assess the content of MTX.

### Cellular uptake studies

JEG-3 cells were used for uptake studies. Briefly, cells were plated overnight in 6-well plates and then treated with culture medium with or without fluorescein-labeled liposomes (Dox-labeled Lipo or Dox-labeled Cy-Lipo) for 4 h. The fluorescence intensity was determined via flow cytometry (FACSCalibur, BD Biosciences, USA). Dox-labeled nanocarrier localization within cells was assessed via confocal laser scanning microscopy (CLSM, FV1200, Olympus, Japan). JEG-3 cells were grown overnight in the confocal dish, followed by treatment for a certain time with Dox-labeled Lipo or Dox-labeled Cy-Lipo-contained culture medium. Finally, cells were fixed with 4% (v/v) paraformaldehyde and nuclei were DAPI stained before testing.

### The mechanism of ENT1-mediated endocytosis

To explore the role of ENT1 in the uptake of Cy grafted liposomes, two kinds of high-affinity substrates of ENT1 were selected for competitive inhibition experiments. Briefly, different concentration (0.2–5.0 μM) of 2′-deoxycytidine or gemcitabine was cultured with the adherent JEG-3 cells for 2 h. Then, the medium was removed, and the fresh medium containing Dox-labeled liposomes was added to the plate. The cells were incubated for another 4 h. Subsequent steps were the same as those in uptake assay.

Endocytosis was studied by adding JEG-3 cells to 6 well-plates and pre-treating them for 30 min with inhibitors of various endocytic pathways including the clathrin-dependent endocytosis inhibitor chlorpromazine (50 μM), the caveolin-dependent endocytosis inhibitor indomethacin (50 μM), the micropinocytosis inhibitor colchicine (10 μM), and quercetin (10 μM) as tools for inhibiting caveolae- or clathrin-independent endocytosis. Then, the medium was removed, and the fresh medium containing Dox-labeled liposomes was added to the plate. The cells were incubated for another 4 h. Subsequent steps were the same as those in uptake assay.

ENT1 regulatory mechanism of JEG-3 cells during the uptake of Cy-Lipo was evaluated on protein and mRNA levels. The JEG-3 cells were added to 6-well plates at 10^5^ cells/well. At 24 h post-plating, 5 μg/mL of Cy-Lipo was added and the cells were cultured for 0, 0.5, 1, 2, 4, 8, 12, and 24 h. At different time point, the cells were collected and a protein extraction kit was used to isolate cytosolic (Beyotime, China) or membrane proteins (Invent Biotechnology, China). Segregated proteins were assessed via western blotting as previously described, with β-actin and cadherin as respective cytosol and membrane controls. Furthermore, JEG-3 cells treated as described above were used for RNA isolation for subsequent RT-PCR as previously described.

### In vitro cytotoxicity

The cytotoxicities of MTX formulations were assessed via MTT assay. The groups were as follows: MTX solution (free MTX), MTX plus DSPE-PEG_2k_-Cy (abbreviated as MTX + Cy-lipid, the molar ratio of Cy-lipid to MTX was about 1:5.0), Lipo@MTX, and Cy-Lipo@MTX. JEG-3 cells or Pricell-0051 cells were added to 96-well plates (1 × 10^4^ cells/well). After 24 h, cells were rinsed using PBS and treated with MTX formulations (0.001–30 μg MTX-equivalent/mL) for 48 h at 37 °C [40]. Viability was assessed by adding 20 μL of MTT (5 mg/mL) per well for 4 h. Next, the medium was removed and formazan crystals were dissolved via the addition of 150 μL of DMSO. A microplate reader (Varioskan Flash 3001, Thermo Fisher Scientific, USA) was then used to assess absorbance at 490 nm.

To investigate the effect of ENT1 on MTX formulations induced cytotoxicity, 2′-deoxycytidine and gemcitabine, as competitive inhibitors of ENT1, were cultured with adherent JEG-3 cells for 2 h. Next, the medium was removed, and the fresh medium containing MTX formulations (20 μg MTX-equivalent/mL) was added to the plate. The cells were incubated for another 48 h. MTT reagent was added as previously described, and absorbance was assessed via microplate reader.

### Cell cycle and apoptosis studies

For cell cycle analysis, JEG-3 cells (10^5^/well) were added to 6-well plates for 24 h and were then treated with culture medium or MTX formulation-contained culture medium at an equivalent MTX concentration (1 μg/mL) for another 24 h. Then, JEG-3 cells were collected (1000 rpm, 5 min) and fixed using 70% ethanol for 8 h at 4℃. After resuspended in PI/RNase Staining buffer for 30 min, the cell cycle was determined with flow cytometry.

Apoptosis of JEG-3 cells was detected using the FITC Annexin V Apoptosis Detection Kit I. The cells (10^5^/well) were seeded in 6-well plates. Following culture for 24 h, cells were treated with culture medium or MTX formulation-contained culture medium at an equivalent MTX concentration (1 μg/mL) for another 24 h. All other protocols were conducted based on the provided directions. The cells were analyzed by flow cytometry.

### Mitochondrial transmembrane potential changes and cell structure damage studies

Mitochondrial transmembrane potential changes induced by various MTX formulations were evaluated by the JC-1 probe. Briefly, JEG-3 cells were added to the confocal dish. Following culture for 24 h, the cells were treated with free MTX, MTX + Cy-lipid, Lipo@MTX, or Cy-Lipo@MTX at an equivalent MTX concentration (1 μg/mL) for 24 h. JC-1 solution was exchanged for cold PBS following two JC-1 staining buffer washes, after which samples were assessed by CLSM at λex (488 nm)/λem (530 nm) for green fluorescence and λex (525 nm)/λem (590 nm) for red fluorescence.

Bio-TEM was applied for observing the cell structure and mitochondrial damage effect induced by MTX formulations. Briefly, JEG-3 cells were seeded in a 6 cm dish. After culturing for 24 h, the cells were treated with free MTX, MTX + Cy-lipid, Lipo@MTX, or Cy-Lipo@MTX at an equivalent MTX concentration (1 μg/mL) for another 24 h. Then, cells were digested, centrifuged, and fixed with 2.5% glutaraldehyde solution at 4 °C for more than 4 h. Finally, the bio-TEM observation was performed after sample preparation.

### In vivo fluorescence studies

BALB/c nude mice were subcutaneously injected with 1 × 10^7^ JEG-3 cells in the flank. When JEG-3 tumors in BALB/c mice were 200–300 mm^3^ in size, IVIS imaging systems (PerkinElmer, USA) were used to assess mice at specified time points following intravenous administration of IR780-labeled Lipo or IR780-labeled Cy-Lipo, respectively. The fluorescence images were collected at pre-treatment or suitable time points (0.5 h, 2 h, 6 h, 12 h, and 24 h) after treatment.

### Pharmacokinetic and biodistribution studies

The advanced Automatic Blood Collection System (Instech, USA) was applied to study the pharmacokinetic feature of various MTX formulations. Female SD rats (200–220 g) were acclimatized at 25 ± 2 °C for 1 week before the experiments. Animals were randomized into 3 groups and each group contained 3 rats. Group I, II, and III were intravenously given free MTX, Lipo@MTX, and Cy-Lipo@MTX respectively at a dose of 10 mg MTX-equivalent/kg body weight. Blood (200 μL) was obtained at 0.25, 0.5, 1, 2, 4, 8, 12, 24, 48, and 72 h through jugular vein after administration. 150 μL of plasma was combined with 450 μL acetonitrile to precipitate proteins, and drug content of samples was analyzed by HPLC. The pharmacokinetic software (DAS 2.0) was used to assess key pharmacokinetic parameters including mean residence time (MRT), area under the curve (AUC), peak plasma concentration (C_max_), half-life (t_1/2_), and time to maximal plasma concentration (T_max_).

JEG-3 tumor-bearing mice were also used for organ distribution studies. Animal groups and dosage were consistent with pharmacokinetic studies. For bio-distribution studies of MTX formulations, three animals per group were sacrificed at 2, 8, and 24 h following treatment, and tumors, lungs, kidneys, hearts, livers, and spleens were isolated, weighed, and frozen. These tissues were then homogenized, spun down for 1 min, rested for 45 min, and combined with 100 µl of 10% trichloroacetic acid solution followed by vortexing for 1 min, adding 5 mL of acetonitrile, and incubating for 10 min. Samples were then spun down for 10 min at 6,000 rpm, and supernatants were isolated, combined with mobile phase, and passed through 0.22 µm membrane filters. Finally, HPLC was applied to estimate the drug content of samples.

### In vivo antitumor studies

BALB/c nude mice were subcutaneously injected with 1 × 10^7^ JEG-3 cells in the flank. When tumors were 50–100 mm^3^ in size, nude mice were intravenously administrated with saline or MTX formulations (free MTX, MTX + Cy-lipid, Lipo@MTX, or Cy-Lipo@MTX, 5 mg MTX-equivalent/kg body weight, each group contained 6 mice) on day 6, 9, 12, 14, 18, and 21. On the 24^th^ day, the mice were sacrificed, and the tumors, as well as the main organs, were excised, weighed, washed three times using saline, and subjected to fixation with 10% neutral buffered formalin. The tumor tissues were collected for H&E, TUNEL, and Ki67 staining. All immunohistochemical sections were observed with a digital scanning microscope imaging system (OCUS-100117, Grundium, Finland). The survival of large tumor-bearing mice (300–500 mm^3^ in size) was analyzed via Kaplan–Meier analysis (each group contained 5 mice).

### In vivo biocompatibility analysis

To assess the hemolytic properties of Lipo@MTX and Cy-Lipo@MTX, 8 mL fresh anticoagulant blood was isolated from the ear vein of white rabbits. Then, the samples were centrifuged and washed with saline until the supernatant was colorless. The red blood cells were diluted with saline for obtaining 2% (v/v) red cell suspensions. Different concentrations of Lipo@MTX or Cy-Lipo@MTX (from 0.01 to 2.0 mg/mL) were then co-incubated with red cell suspensions for 1 h at 37 °C. Simultaneously, equal volume of ultrapure water was selected as positive control (hemolysis rate: 100%). After incubation, samples were centrifuged, and the absorbance of the supernatant was measured at 414 nm using a microplate reader.

Healthy mice were administered the Lipo@MTX or Cy-Lipo@MTX dispersion via tail vein (10 mg MTX-equivalent/kg body weight every three days) for five times (each group contained 3 mice). Saline was injected as a control. After the administration, blood biochemical indices were measured. During the in vivo antitumor studies, the body weight and activity status of mice in each group were monitored for evaluating the side effects of the MTX formulations. After in vivo antitumor studies, the primary organs of each group were harvested for H&E staining.

### Statistical analysis

All data were assessed with SPSS v17.0 (IBM Inc., IL, USA) and expressed as the mean ± SD. P < 0.05 was the significance threshold.

## Results and discussion

### Specific expression of ENT1 on JEG-3 cells

**Fig. 2:**
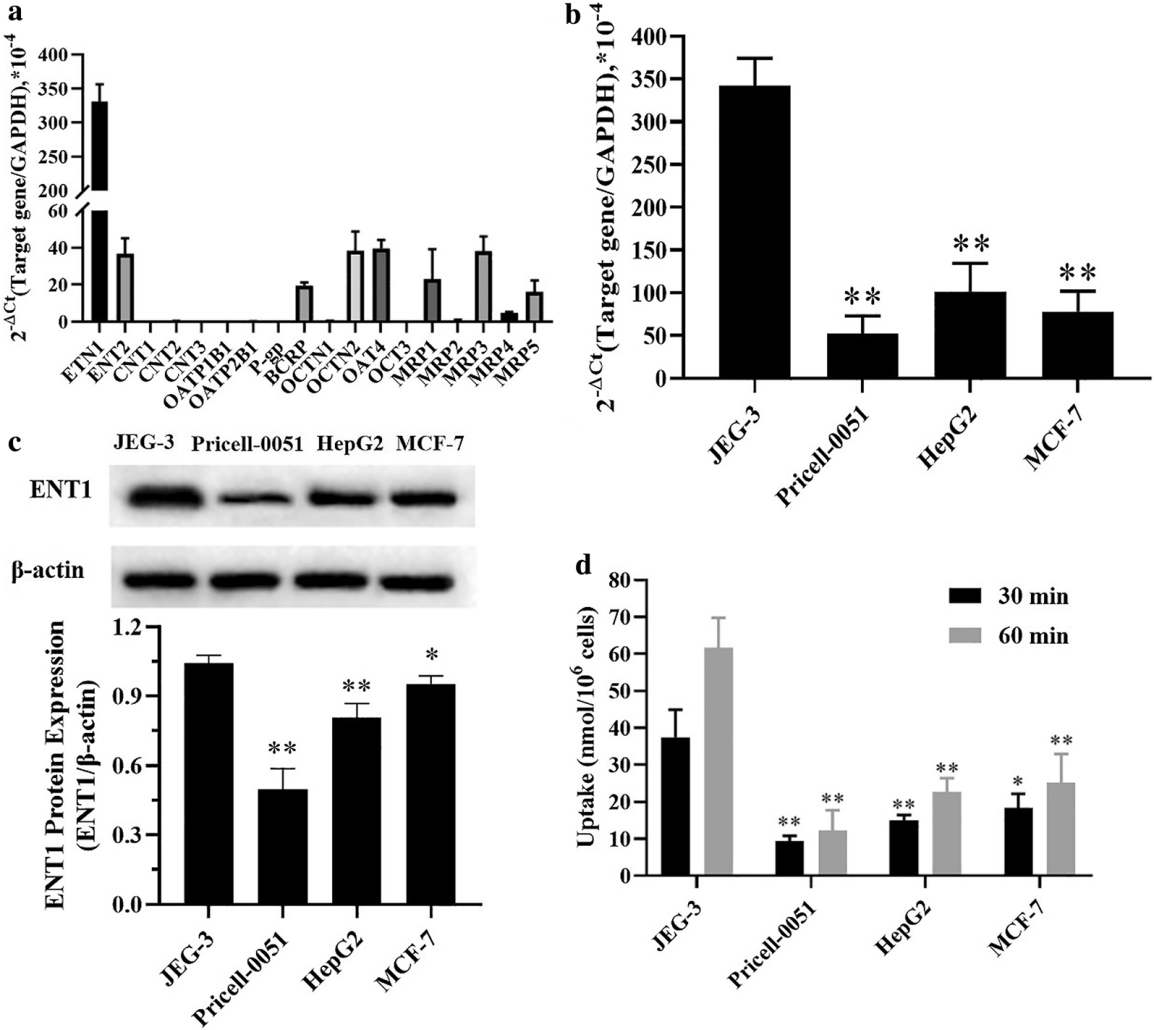
mRNA expression of multiple transporters in JEG-3 cells (**a**). mRNA (**b**) and protein (**c**) expression of ENT1 transporter in Pricell-0051, HepG2, MCF-7, and JEG-3 cells. The uptake of cytarabine by the above cells within a certain period of time (**d**). *P < 0.05, **P < 0.01 *vs* JEG-3 cells

To make a breakthrough in the dilemma of choriocarcinoma therapy and find a target membrane protein for specific drug delivery, transporter mRNA expression in human choriocarcinoma cells (JEG-3) was analyzed. The results indicated that the mRNA expression of human equilibrative nucleoside transporter 1 (ENT1) in JEG-3 cells was significantly higher than that of the other cell membrane transporters, such as concentrative nucleoside transporters (CNTs), organic anion transporters (OATs), organic cation transporters (OCTs), carnitine/organic cation transporters (OCTNs), multidrug resistance-associated protein (MRPs), P-glycoprotein (P-gp), and breast cancer resistance protein (BCRP). The gap in the expression between ENT1 and these other transporters was an order of magnitude (Fig. 2a). The mRNA level of ENT1 in JEG-3 cells was approximately 6.3 times of that in normal human placental trophoblast cells (Pricell-0051), and significantly higher than that in human liver cancer cells (HepG2) and human breast cancer cells (MCF-7) (Fig. 2b). Similarly, the expression of ENT1 protein in JEG-3 cells was significantly higher than that in Pricell-0051, HepG2, and MCF-7 cells (P < 0.05) (Fig. 2c). Furthermore, our group explored the uptake of the nucleoside analogue cytarabine (a substrate of ENT1) by the above four cell lines (Fig. 2d). The transport of Cy into cells was a time-dependent process, and the amount taken up by JEG-3 cells was significantly higher than that taken up by Pricell-0051, MCF-7, and HepG2 cells after 30 min and 1 h (P < 0.05). The above results suggested the specific expression of ENT1 in JEG-3 cells, which made it a potential target for choriocarcinoma therapy.

### Preparation and characterization of Lipo@MTX and Cy-Lipo@MTX

Methotrexate (MTX) is the most important first-line drug for the treatment of trophoblastic diseases. It is a polar molecule with a structure similar to that of folic acid and can enter the cell in two ways: through the folic acid transporter and by passive diffusion (Additional file 1: Fig. S1b). The first transport pathway has a higher affinity for folic acid and a low affinity for MTX. Additionally, it is difficult for the polar small molecule MTX to penetrate cell membranes [8]. Moreover, patients with trophoblastic tumors have serious resistance to MTX [41]. Conventional doses of MTX are usually ineffective, so higher doses or combination chemotherapy are required. However, these factors will lead to longer treatment times, more side effects, and a higher risk of treatment failure [42]. The lack of specific distribution in lesion sites and the low transportation efficiency of MTX are key scientific issues in the treatment of trophoblast-related diseases (as displayed in Additional file 1: Fig. S1b).

**Fig. 3:**
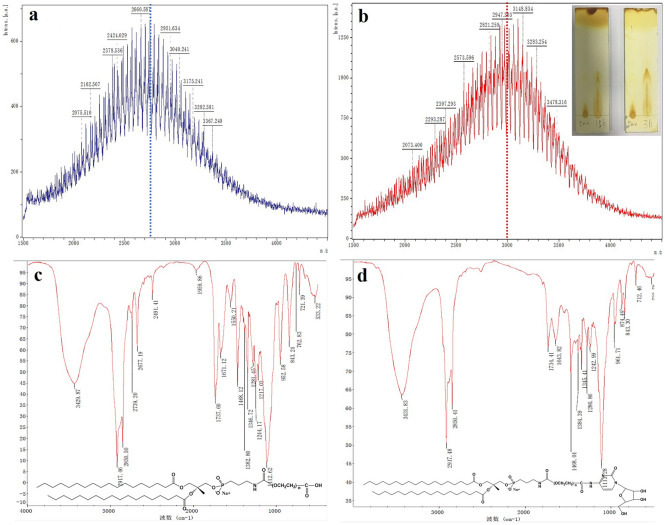
MALDI-TOF–MS spectrum of DSPE-PEG_2k_-COOH (**a**) and DSPE-PEG_2k_-Cy (**b**). Thin layer chromatography was applied to monitor the synthesis reaction of DSPE-PEG_2k_-Cy (**b** insert). FT-IR spectrum of DSPE-PEG_2k_-COOH (**c**) and DSPE-PEG_2k_-Cy (**d**)

Liposomes are the most common nanocarriers used for targeted drug delivery systems with biodegradability, low toxicity, and low immunogenicity [43, 44]. Moreover, liposomes have several advantages to overcome obstacles in cellular uptake and improve the payload biodistribution [45]. Many liposome formulations have been approved for clinical cancer therapy, such as Doxil®, Onivyde®, and Vyxeos® [46]. In this study, liposomes were selected as the drug carrier for intracellular delivery of MTX. First, a kind of Cy-conjugated distearoylphosphatidylethanolamine-polyethylene glycol (DSPE-PEG_2k_-Cy, abbreviated as Cy-lipid) was synthesized through a condensation reaction between the amine of Cy and the active carbonyl group of the carboxyl-terminated PEG-lipids (DSPE-PEG_2k_-COOH). In the MALDI-TOF–MS spectrum, the m/z peak of DSPE-PEG_2k_-COOH is approximately 2750 (Fig. 3a) and that of DSPE-PEG_2k_-Cy is approximately 2995 (Fig. 3b). The difference in molecular weight between DSPE-PEG_2k_-COOH and DSPE-PEG_2k_-Cy is 245, which is approximately the molecular weight of Cy (MW = 243.23). The new peak at 1643.82 cm^−1^ in the FT-IR spectra was caused by the absorption of  −C=O bond in pyrimidone ring of DSPE-PEG_2k_-Cy, and the characteristic absorption of −C-H adjacent to oxygen in the epoxy group of Cy was 874.49 cm^−1^ (Fig. 3c, d). The MALDI-TOF–MS and FT-IR results showed the successful synthesis of DSPE-PEG_2k_-Cy.

**Fig. 4:**
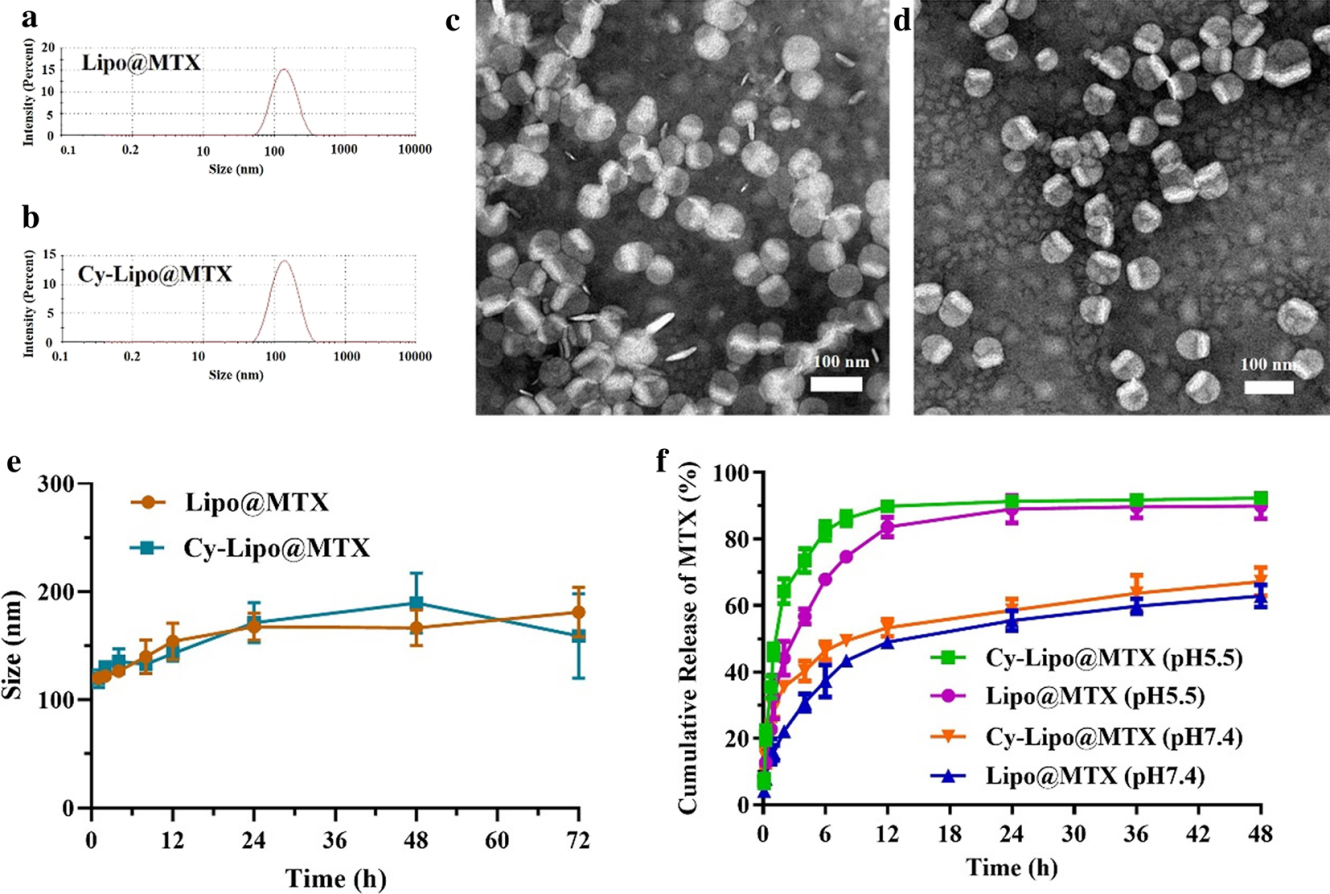
Particle size distribution of Lipo@MTX (**a**) and Cy-Lipo@MTX (**b**). TEM images of Lipo@MTX (**c**) and Cy-Lipo@MTX (**d**). Dynamic light scattering profiles of Lipo@MTX and Cy-Lipo@MTX in PBS solution containing 10% FBS for 72 h (**e**). Release profiles of Lipo@MTX and Cy-Lipo@MTX in buffer at different pH values (**f**)

MTX-loaded liposomes were prepared by the thin-film hydration method combined with high-pressure homogenization. The prepared Lipo@MTX and Cy-Lipo@MTX were approximately 120 nm in diameter (Fig. 4a, b) with perfect dispersion as observed in the TEM images (Fig. 4c, d). The lyophilized Cy-Lipo@MTX had a smooth appearance without collapse after using 5% mannitol (w/v) as cryoprotectant (Additional file 1: Fig. S2a). The lyophilized Cy-Lipo@MTX had excellent re-dispersion in PBS with shake, and there was no aggregation in the TEM image (Additional file 1: Fig. S2b, c). After being stored at room temperature for five and nine months, the particle size of Cy-Lipo@MTX could maintain at approximately 150 nm (Additional file 1: Fig. S2d, e). The above results showed that the lyophilized formulation had good storage stability. The content of Cy-lipid in Cy-Lipo@MTX was calculated to be approximately 13.51%. The DL% and EE% of Lipo@MTX (8.92 ± 1.31% and 44.61 ± 6.59%, respectively) were similar to those of Cy-Lipo@MTX (9.46 ± 1.01% and 48.53 ± 2.54%, respectively). Next, DSC was applied to analyze the crystal form of the drug in Cy-Lipo@MTX (Additional file 1: Fig. S3). It was observed that the pure crystalline MTX exhibited a peak at 123.0 °C and this characteristic peak of MTX was absent in the DSC curve of Cy-Lipo@MTX, which suggested that the MTX had been converted to its amorphous form after encapsulated by liposomes [47].

The ability of MTX-loaded liposomes to accumulate within tumors is dependent upon their stability within the circulation. We thus tested the stability of MTX formulations for 72 h in PBS supplemented with 10% FBS (pH 7.4). Over this period, there was a slight increase in the particle size of Cy-Lipo@MTX and Lipo@MTX. The particle size maintained below 200 nm (Fig. 4e), and there was no precipitation in the dispersion. The high stability of Cy-Lipo@MTX and Lipo@MTX may be due to the PEG chains exhibiting steric hindrance and reducing serum protein interference [48]. This feature is critical for Cy-Lipo@MTX in cancer therapy to increase the distribution to the tumor site through the enhanced permeability and retention (EPR) effect.

### In vitro drug release

The in vitro drug release patterns of Lipo@MTX and Cy-Lipo@MTX were evaluated in buffer at pH values of 7.4 or 5.5. According to the results of DSC, MTX exists in an amorphous form in liposomes, thus it is easier to dissolve in aqueous solutions. As shown in Fig. 4f, MTX release from Lipo@MTX and Cy-Lipo@MTX was promoted by a decrease in the environmental pH value. This is because the solubility of MTX in acidic solutions is greater than that in neutral solutions [49]. At pH 7.4, Lipo@MTX and Cy-Lipo@MTX released 22.10 ± 1.3% and 35.53 ± 3.27% MTX, respectively, within 2 h. In comparison, 44.11 ± 8.76% and 64.27 ± 6.52% MTX was released within 2 h in an acidic environment (pH 5.5). The release rates of Lipo@MTX in the pH 7.4 and pH 5.5 environments were 62.83 ± 5.86% and 89.96 ± 6.67% after 48 h, respectively, and the release rates of Cy-Lipo@MTX in these two environments were 67.17 ± 7.39% and 92.41 ± 1.52% after 48 h, respectively. The release profiles of the two nano-formulations at the same pH were similar, indicating that Cy surface functionalization would not influence the release of MTX. The pH-responsive drug release pattern of Cy-Lipo@MTX might be facilitated to reduce the burst release and leakage of MTX during body circulation. The stability and in vitro drug release studies illustrated that the encapsulated MTX could not only achieve long-term circulation of the drug in the body but also realize specific drug release in the acidic tumor environment. These two properties are essential for the antitumor nano-formulations, as they can reduce toxicity and increase efficacy [50, 51].

### In vitro cellular uptake studies

**Fig. 5:**
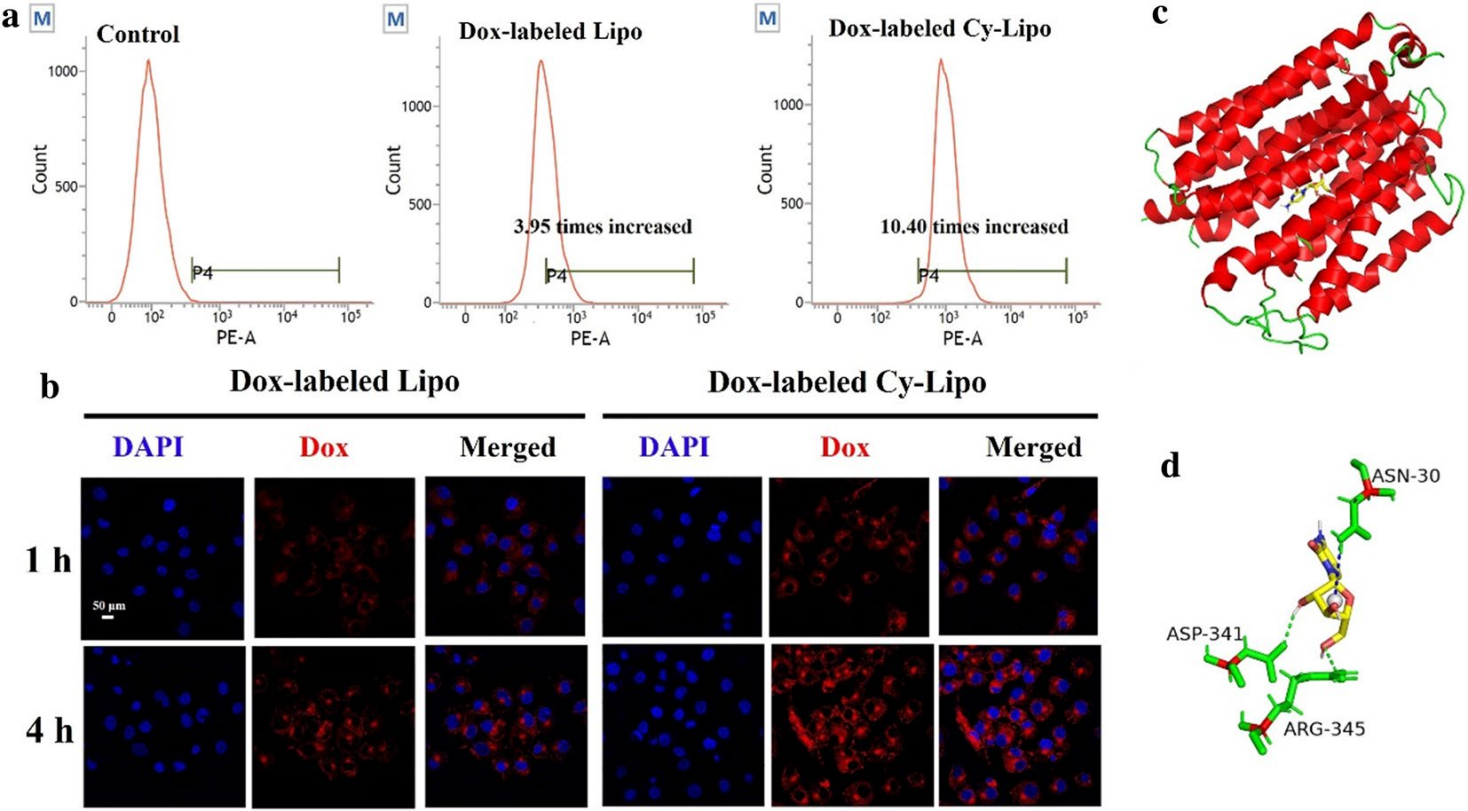
Flow cytometry profiles of JEG-3 cells that were incubated with culture medium, Dox-labeled Lipo or Dox-labeled Cy-Lipo for 4 h (**a**). Confocal images of JEG-3 cells incubation with Dox-labeled Lipo or Dox-labeled Cy-Lipo for 1 h and 4 h. The nucleus of cells was stained with DAPI (blue) (**b**). Computational ENT1 docking analysis. Docking of Cy inside the special binding sites of ENT1 (**c**) and the residue interaction between ENT1 and Cy (**d**)

The fluorescence intensity of Dox-labeled Lipo-incubated JEG-3 cells was 3.95 times stronger than that of the control group after incubation for 4 h, while that of Dox-labeled Cy-Lipo increased 10.40-fold (Fig. 5a). The above results revealed that modification with Cy increased the uptake of the liposomes by JEG-3 cells. Next, CLSM was applied to observe the fluorescence localization within JEG-3 cells at different time points. As shown in Fig. 5b, the red fluorescence of Dox-labeled Cy-Lipo was mainly distributed in the cell membrane and cytoplasm after incubation for 1 h. In comparison, low-intensity red fluorescence could be observed in the Dox-labeled Lipo-incubated JEG-3 cells at 1 h. After co-incubation for 4 h, the red fluorescence intensity of Dox-labeled Cy-Lipo-treated cells was still significantly stronger than that of the Dox-labeled Lipo group. CLSM observations confirmed the role of Cy in increasing the cellular uptake of Cy-Lipo. Mechanistically, it was speculated that Cy could bind specifically to ENT1 as a substrate and mediate the entry of Cy-Lipo into the cells through a certain effect, which would be explored in the following studies.

### Computational ENT1 docking analysis

To explore the Cy binding properties in the context of ENT1 transport, we conducted molecular dynamic simulations. From a thermodynamic perspective, stable systems are denoted by negative free energy (ΔG < 0) [52]. The ΔG between Cy and ENT1 was − 6.5 kcal/mol, which indicated good binding effect between the two molecules. Two hydrogen bonds were formed between the hydroxyl of Cy and ASP-341 and ARG-345 of ENT1 (Fig. 5c, d). Moreover, there were hydrophobic interactions between Cy and ASN-30 of ENT1. The docking analysis results demonstrated that these three interaction sites were all on the furan ring of Cy and the amide group formed between Cy and DSPE-PEG_2k_-COOH was on the pyrimidine ring of Cy. Thus, the DSPE-PEG_2k_ chain of DSPE-PEG_2k_-Cy had little influence on the molecular docking process between Cy and ENT1. Thus, ENT1 could specifically recognize and transport Cy-conjugated nanodrugs into JEG-3 cells.

### Assessment of the mechanisms of ENT1-mediated endocytosis

**Fig. 6:**
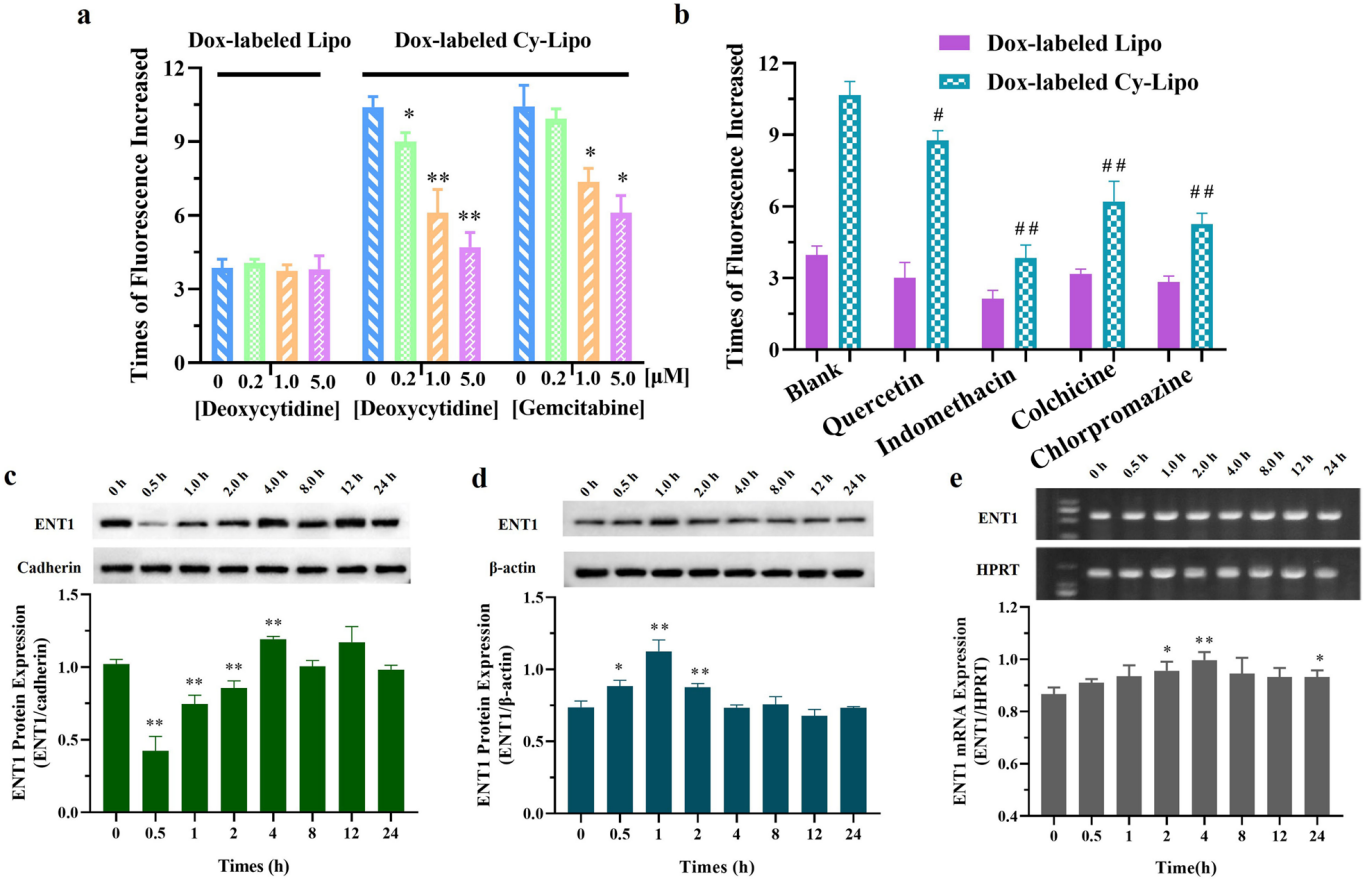
Influence of ENT1 substrates (**a**) or endocytosis inhibitors (**b**) on cellular uptake of Dox-labeled Lipo or Dox-labeled Cy-Lipo. *P < 0.05, **P < 0.01, *vs* cells without pre-treatment with deoxycytidine or gemcitabine. ^#^P < 0.05, ^##^P < 0.01, *vs* cells without pre-treatment with endocytosis inhibitors**.** The variations of membrane (**c**) and cytosol (**d**) ENT1 protein expression after treatments with Cy-Lipo over 24 h. Analysis of ENT1 mRNA in JEG-3 cells after incubation with Cy-Lipo over 24 h (**e**). *P < 0.05, **P < 0.01, *vs* cells without treatment with Cy-Lipo

To further clarify the mechanism of ENT1-mediated Cy-Lipo endocytosis into cells, competitive inhibitors (deoxycytidine and gemcitabine) of ENT1 and endocytosis inhibitors were applied in the cellular uptake studies. The results showed that deoxycytidine could significantly reduce the uptake of Dox-labeled Cy-Lipo by JEG-3 cells in a concentration-dependent manner (P < 0.05) but had less of effect on Dox-labeled Lipo when the inhibitor concentration was between 0.2 μΜ and 5.0 μΜ (Fig. 6a). These results were because the structure of deoxycytidine was similar to that of Cy. Deoxycytidine can occupy the binding sites between Cy and ENT1, thus hindering the binding of ENT1 and Cy and ultimately reducing the endocytosis of Dox-labeled Cy-Lipo. Similarly, gemcitabine, which is a cytosine derivative, also significantly influenced the uptake of Dox-labeled Cy-Lipo at concentrations of 1.0 μM and 5.0 μM (P < 0.05). These results indicated that the uptake of Cy-modified formulations was highly correlated with the function of ENT1. Then, we further examined the influence of endocytosis inhibitors on the uptake of Cy-modified nano-formulations. The caveolin-mediated endocytosis inhibitor indomethacin impacted the uptake of Dox-labeled Cy-Lipo to the greatest extent and the clathrin-mediated endocytosis inhibitor chlorpromazine showed the next most substantial inhibition among all endocytosis inhibitors (Fig. 6b). A reduced effect was observed for the micropinocytosis inhibitor colchicine compared to indomethacin and chlorpromazine. Quercetin, which inhibits caveolin/clathrin-independent endocytosis, had a certain inhibitory effect on the endocytosis of Dox-labeled Cy-Lipo. The above results showed that binding with ENT1 and endocytosis were two important processes for ENT1-mediated Cy-Lipo uptake by JEG-3 cells.

**Fig. 7:**
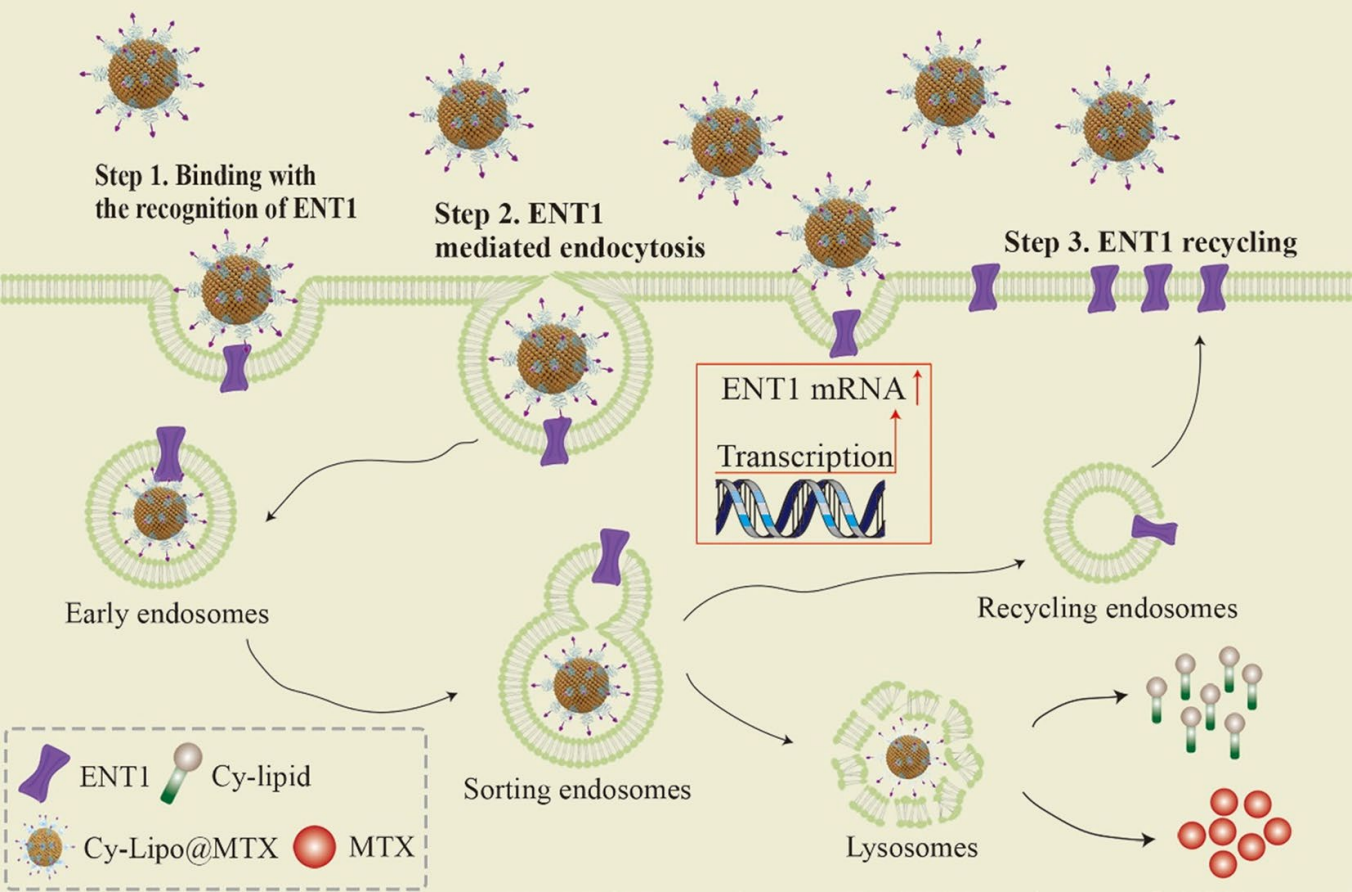
The schematic illustration presenting the ENT1-mediated endocytic cycle mechanism

To explore whether ENT1 was involved in the process of endocytosis, our group assessed time-dependent alterations in ENT1 protein levels via western blotting analysis. We found a significant decrease in membrane protein level in JEG-3 cells following 30 min of exposure to Cy-Lipo compared to the control level; however, the protein level recovered with time, and the level returned to normal between 0.5 h and 4 h (Fig. 6c). In the cytoplasm, ENT1 protein level showed an increasing trend from 0 to 2 h and then returned to its original level (Fig. 6d). Furthermore, the mRNA level of ENT1 increased from 0.5 h to 4 h during the uptake process, and there was a significant difference in the expression at 2 h and 4 h compared to the control level (P < 0.05) (Fig. 6e). These data indicated that part of the ENT1 protein on the membrane entered the cytoplasm along with the endocytic vesicles. The recovery of protein level over time may be due to partial endocytosed transporter recycling and additional transporter synthesis, as evidenced by the increased expression of mRNA. A schematic illustration presenting the ENT1-mediated endocytic cycle mechanism was displayed in Fig. 7.

### In vitro cytotoxicity

**Fig. 8:**
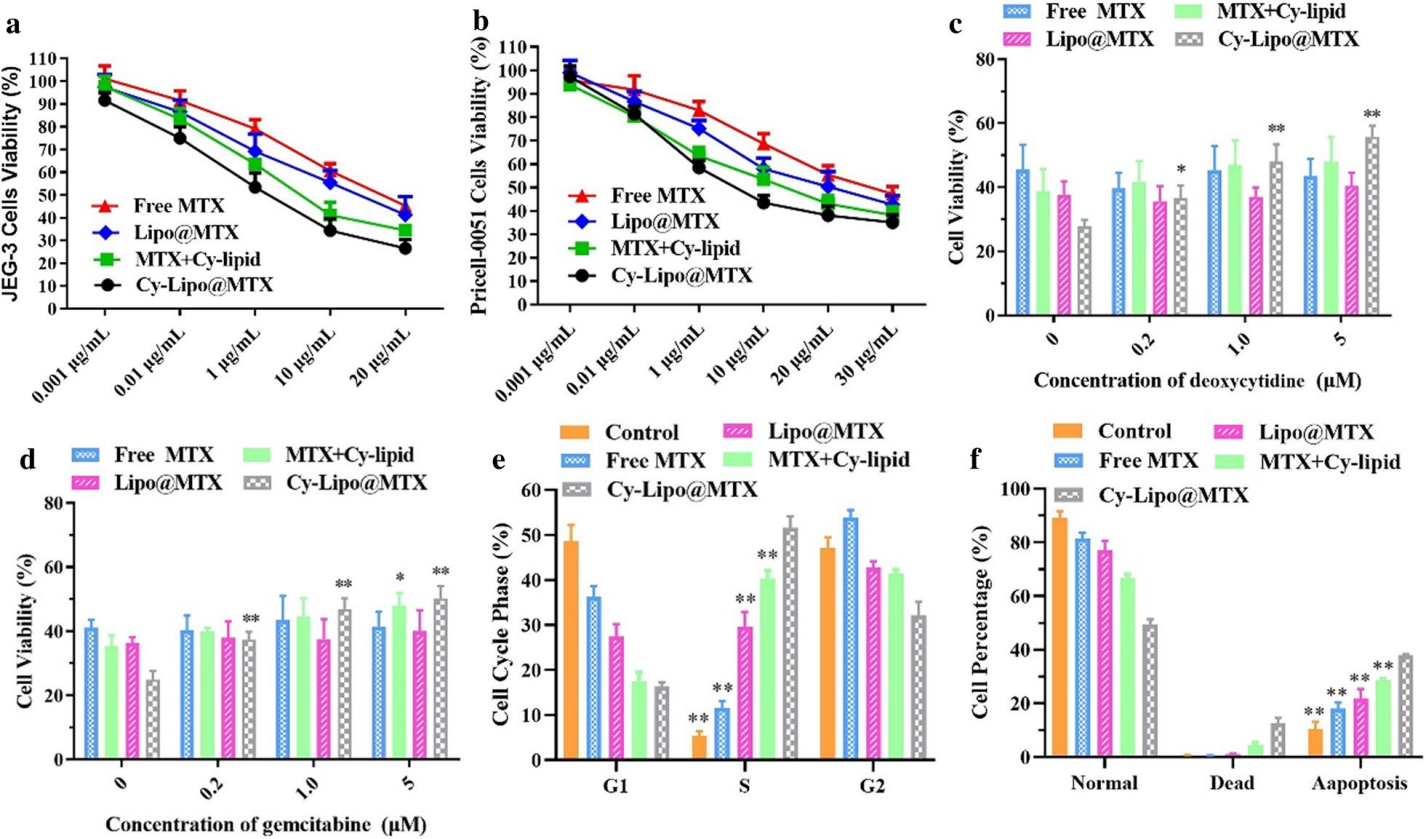
The viability of JEG-3 (**a**) and Pricell-0051 cells (**b**) after being treated with free MTX, physical mixture of MTX and Cy-lipid, Lipo@MTX, or Cy-Lipo@MTX for 48 h during a certain drug concentration range. The effect of ENT1 competitive inhibitors on MTX formulations induced cytotoxicity (**c**, **d**). *P < 0.05, **P < 0.01, *vs* cells without pre-treatment with deoxycytidine or gemcitabine. Cell cycle distribution induced by culture medium or MTX formulation-contained medium for 24 h (**e**). Quantitative analysis of apoptosis after treating JEG-3 cells with culture medium or MTX formulation-contained medium for 24 h (**f**). **P < 0.01, *vs* Cy-Lipo@MTX group

The cytotoxicities of free MTX, a physical mixture of Cy-lipid and MTX, Lipo@MTX, and Cy-Lipo@MTX were evaluated in JEG-3 and Pricell-0051 cells for 48 h (Fig. 8a, b). All of the MTX formulations displayed dose-dependent cytotoxicity to the JEG-3 and Pricell-0051 cell lines. The half maximal inhibitory concentration (IC_50_) of each MTX formulation in normal cells (Pricell-0051) was greater than that of JEG-3 cells, indicating that JEG-3 was more sensitive to MTX (Table 1). For that reason, MTX has been selected as one of the most important first-line drugs for choriocarcinoma therapy. The mean IC_50_ of Lipo@MTX in JEG-3 cells (12.84 μg/mL) was smaller than that of free MTX (19.55 μg/mL). This was because the polar small molecule MTX were more difficult to penetrate cell membranes than the nano-formulations [8], and JEG-3 cells could take up Lipo@MTX through endocytosis and membrane fusion. Cy-conjugated lipids, which were formed through the link between the amino terminus of Cy and the carboxyl terminus of the lipid, were demonstrated to have improved antitumor activity in tumor cells compared to free Cy [53]. Thus, the Cy-lipid prepared in our research may be able to play a synergistic role against choriocarcinoma. As displayed in Table 1, the mean IC_50_ of the MTX + Cy-lipid group (5.57 μg/mL) was smaller than that of both the free MTX group and Lipo@MTX group, which confirmed the synergistic antitumor effect of MTX and Cy-lipid. The cytotoxicity induced by Cy-Lipo@MTX in JEG-3 cells (mean IC_50_ = 1.51 μg/mL) was higher than that induced by free MTX, MTX + Cy-lipid, and Lipo@MTX. These results showed that the modification of Cy augmented the antitumor cytotoxicity of MTX-loaded liposomes, potentially due to the increased uptake of the nanodrugs by JEG-3 cells. Our group further studied the role of ENT1 in Cy-Lipo@MTX-induced cytotoxicity with competitive inhibition experiments. As displayed in Fig. 8c, d, pre-treatment of deoxycytidine and gemcitabine (at concentrations between 0.2 and 5.0 μM) significantly reduced the toxicity of Cy-Lipo@MTX to JEG-3 cells. This result was consistent with the cellular uptake results in which the competitive inhibitor was able to inhibit the function of ENT1 and reduce the uptake of Cy-Lipo@MTX into cells. In comparison, deoxycytidine and gemcitabine had little effect on the cytotoxicity of free MTX or Lipo@MTX in JEG-3 cells.

**Table 1:** IC_50_ values of MTX formulations in JEG-3 cells and Pricell-0051 cells

IC_50_ (μg/mL)	JEG-3					Pricell-0051			
	free MTX	MTX + Cy-lipid		Lipo@MTX	Cy-Lipo@MTX	free MTX	MTX + Cy-lipid	Lipo@MTX	Cy-Lipo@MTX
Mean	19.55*	5.57	12.84*		1.51	32.18*	13.81	25.67	4.08
SD	1.73	1.92	4.90		0.88	6.84	9.27	17.57	2.56

^*^P < 0.05, *vs* Cy-Lipo@MTX group

### Cell cycle arrest and cell apoptosis-induced effects

To assess how the MTX formulations inhibit the cell cycle, flow cytometry was applied to monitor cell cycle progression. JEG-3 cells were treated with or without MTX formulation-contained medium for 24 h. As displayed in Fig. 8e and Additional file 1: Fig. S4, free MTX, MTX + Cy-lipid, Lipo@MTX, and Cy-Lipo@MTX specifically arrested 11.59 ± 1.53%, 40.39 ± 1.73%, 29.60 ± 3.29%, and 51.66 ± 2.53% of JEG-3 cells at the S phase of the cell cycle, which was a significant increase relative to the control cells (5.44 ± 0.97%). The results demonstrated that all MTX formulations inhibited the proliferation of JEG-3 cells by inducing S phase arrest to different extents, with Cy-Lipo@MTX exhibiting superior S phase arrest (P < 0.05). This could be attributed to the enhanced uptake and synergistic effects of MTX and Cy-lipid in Cy-Lipo@MTX-treated JEG-3 cells. Moreover, the flow cytometry data (Fig. 8f and Additional file 1: Fig. S5) showed that the apoptotic percentages of JEG-3 cells after treatment with free MTX, MTX + Cy-lipid, Lipo@MTX, and Cy-Lipo@MTX were 18.03 ± 2.25%, 28.69 ± 0.48%, 21.76 ± 3.65%, and 38.00 ± 1.36%, respectively, which were much higher than that of the control group (10.39 ± 2.78%) (P < 0.05). Additionally, the proportion of apoptotic cells in the Cy-Lipo@MTX treatment group was significantly higher than that in the other treatment groups (P < 0.05). All of the above cytotoxicity experiments confirmed that Cy-Lipo@MTX had a more powerful tumor suppression effect.

### Mitochondrial transmembrane potential change and cell structure damage

**Fig. 9:**
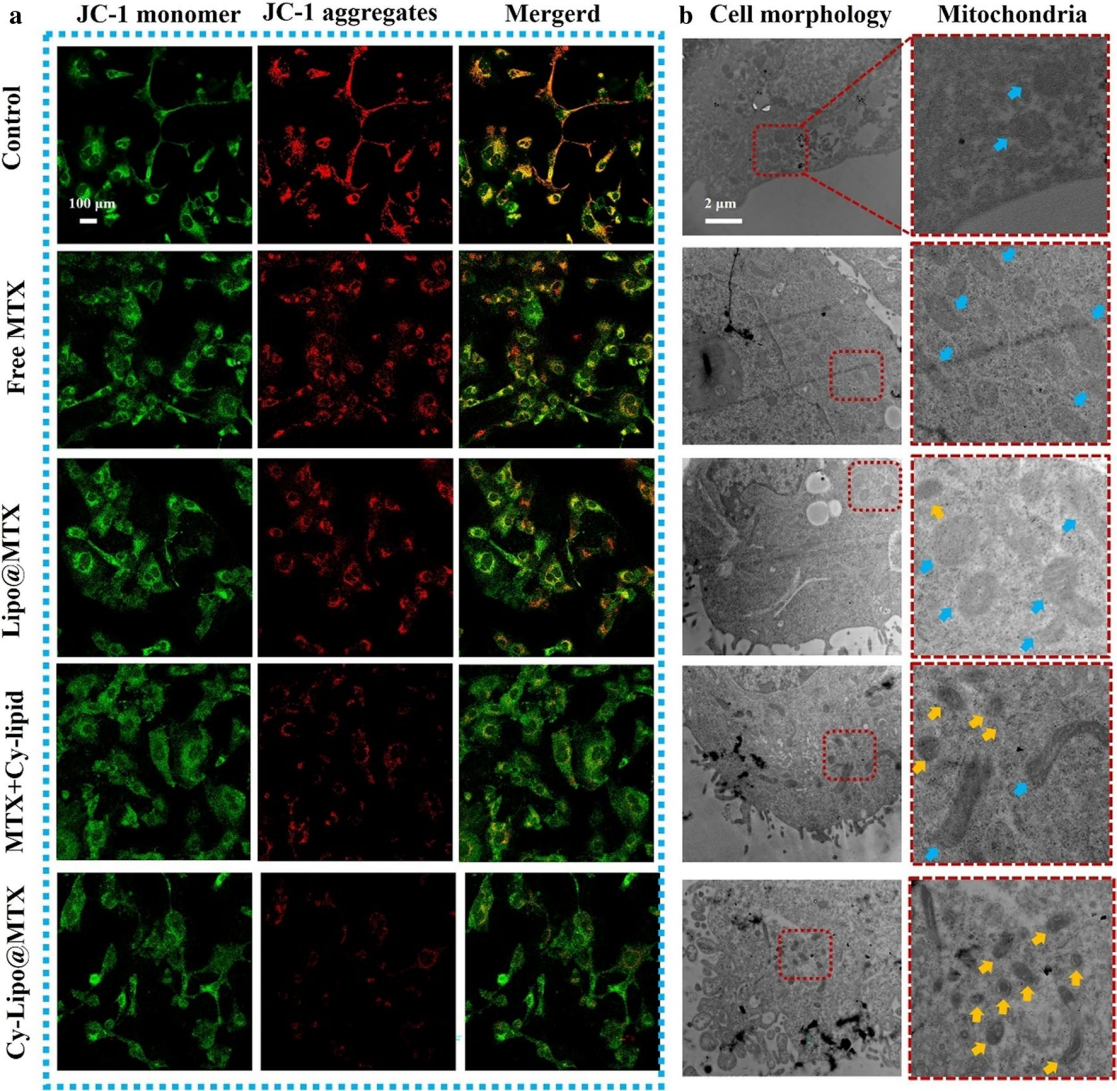
CLSM image of JEG-3 cells after incubation with various MTX formulations for 24 h. Red and green fluorescence represent the JC-1 aggregates and JC-1 monomer, respectively (**a**). The scale bar is 100 μm. Bio-TEM evaluation of the destructive effect caused by MTX formulations on JEG-3 cells, blank culture medium as control (**b**). The red box area is enlarged on the right, the blue arrows indicate the normal mitochondria, and the yellow arrows indicate the atrophied mitochondria

The process of cell apoptosis is often accompanied by a change in mitochondrial transmembrane potential (MMP) [54]. The effect of various MTX formulations on mitochondrial damage in JEG-3 cells was evaluated by measuring the MMP with JC-1 probes. When the MMP level is low, JC-1 cannot accumulate within the mitochondrial matrix but instead exists as a green fluorescent monomer. When JC-1 aggregates, it yields a red fluorescent signal. In CLSM studies (Fig. 9a), there was substantial red fluorescence in the control group, which notably decreased in the free MTX and Lipo@MTX groups. The above results indicated that free MTX and Lipo@MTX had a certain destructive effect on the MMP in JEG-3 cells. The MTX + Cy-lipid-treated tumor cells had a weaker red fluorescence intensity than that in the free MTX and Lipo@MTX groups, which confirmed that MTX and Cy-lipid had a synergistic damaging effect on the MMP. Excitingly, after treatment with Cy-Lipo@MTX, the red fluorescence in JEG-3 cells almost disappeared, indicating the strongest MMP damage among the four MTX formulations. Mitochondrial injury directly affects cell energy metabolism and ultimately induces cell apoptosis. The above research results explained the strongest apoptosis-inducing effects of Cy-Lipo@MTX among the MTX formulations.

To observe the influence of MTX formulations on the mitochondria and cell structure more intuitively, a bio-TEM study was applied. As displayed in Fig. 9b, in the control and free MTX-treated groups, the cell structure was intact and the cell boundary was clearly visible. These mitochondria exhibited a full shape without shrinkage or aggregation (the blue arrows indicate normal mitochondria). After treatment with Lipo@MTX and MTX + Cy-lipid, the tumor cells began to lyse, and cell debris appeared around the cells, which was accompanied by mitochondrial atrophy (the yellow arrows indicate atrophied mitochondria). In the Cy-Lipo@MTX-treated group, the cytoskeleton disintegrated, and significant cell debris was evident at the cell margin, which indicated whole cell destruction. Moreover, a large number of atrophied mitochondria gathered inside the Cy-Lipo@MTX-treated cells. All cell-level studies implicated the potential of Cy-Lipo@MTX against JEG-3 tumor cells.

### Tumor accumulation, pharmacokinetic study and in vivo biodistribution

**Fig. 10:**
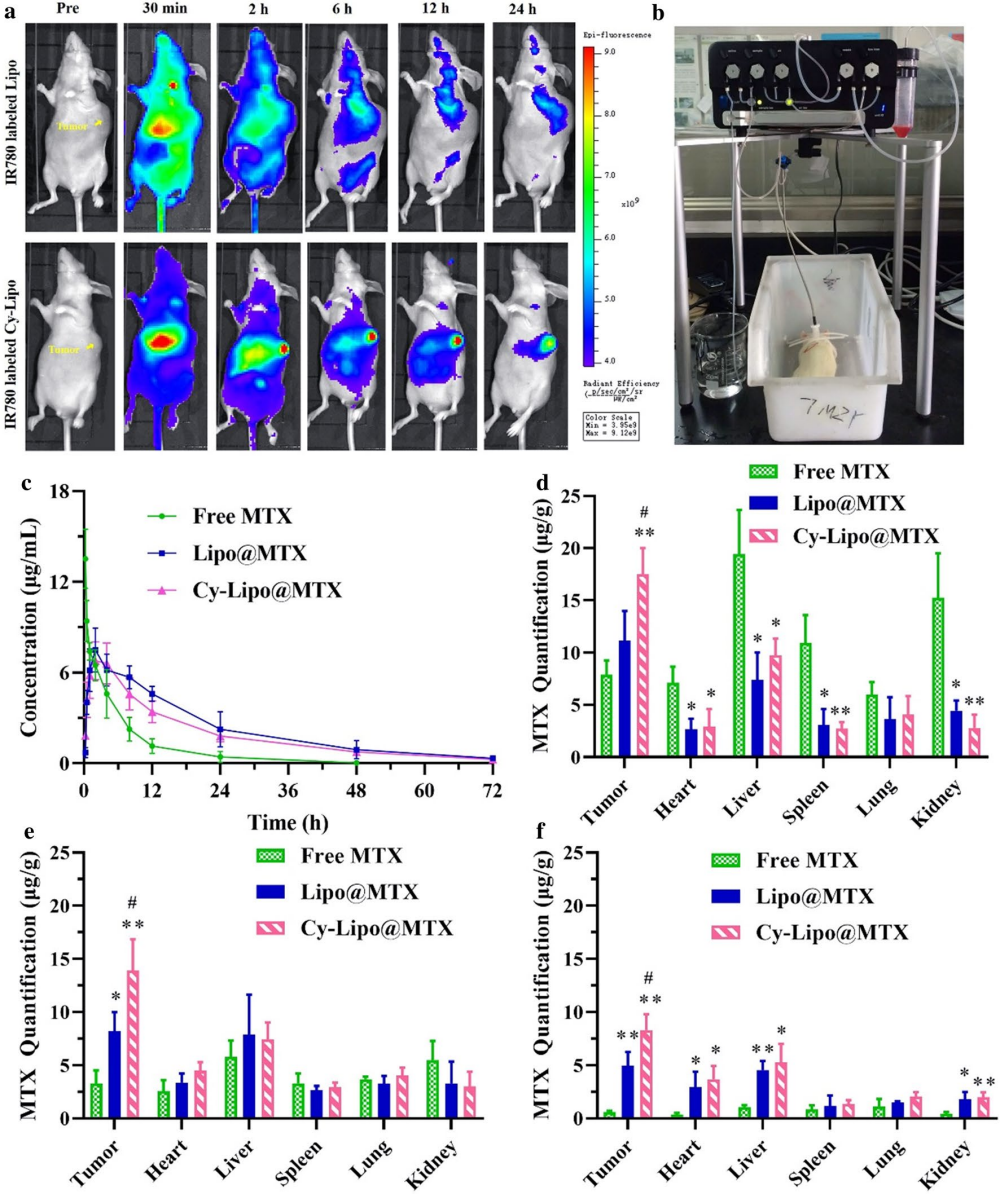
In vivo distribution of IR780-labeled Lipo or IR780-labeled Cy-Lipo in major organs of choriocarcinoma tumor-bearing nude mice during 24 h (**a**). Blood collection from rats by automatic blood sampling device in pharmacokinetic research (**b**). Drug-time curves of MTX formulations in SD rats after intravenous administration (**c**). Biodistribution of MTX in mice at 2 h (**d**), 8 h (**e**), and 24 h (**f**) after intravenous injection of MTX formulations. *P < 0.05, **P < 0.01, *vs* free MTX. ^#^P < 0.05, *vs* Lipo@MTX

Herein, the tumor-accumulating properties of IR780-labeled liposomes were evaluated in choriocarcinoma xenograft nude mice. As seen in Fig. 10a, 30 min after tail vein injection of IR780-labeled Lipo, the fluorescence was systemically distributed, with the strongest fluorescence intensity in the liver. The distribution of IR780-labeled Lipo at the tumor site was not different from that in the other normal tissues except the liver. After 24 h, the fluorescence signal of IR780-labeled Lipo in normal tissues disappeared. However, there was still a fluorescence signal in the proximal tumor site. The fluorescence signal indicated the retention of IR780-labeled Lipo in this region due to the EPR effect of the nano-sized liposomes. In the IR780-labeled Cy-Lipo-treated mice, the fluorescence intensity of the tumor site was significantly stronger than that of other organs after 2 h, and the fluorescence of the tumor site could be retained for 24 h. In comparison, the fluorescence intensity of the tumor in the IR780-labeled Cy-Lipo group was significantly stronger than that of the IR780-labeled Lipo group after 2 h. Due to the specific binding effect between Cy and ENT1 and the enhanced endocytosis effect mediated by ENT1, IR780-labeled Cy-Lipo exhibited better tumor targeting and aggregation effects than IR780-labeled Lipo. The targeting effect of Cy-Lipo would be conducive to the accumulation of more drugs at the tumor site to exert a more powerful antitumor effect with fewer side effects.

In vivo experiments are indispensable for evaluating novel nanodrugs. For the pharmacokinetic studies, an automatic blood collection system was utilized to explore the in vivo processes of the various MTX formulations (Fig. 10b). The sampling process in our research resulted in less damage to the animals and a reduced impact on drug circulation. Drug-time curves and pharmacokinetic parameters were displayed in Fig. 10c and Table 2. Long-circulating liposomes have a certain degree of stability, and they cannot release MTX immediately when they enter the blood circulation. The lipid shell delayed the release of MTX in the body, so a short absorption phase appeared in the drug-time curve of MTX-loaded liposomes [39, 55]. The plasma MTX concentration after free MTX administration was 13.52 ± 1.96 μg/mL after 0.25 h, followed by sharp reductions after 4 h (4.59 ± 1.61 μg/mL), 12 h (1.14 ± 0.49 μg/mL), and 24 h (0.43 ± 0.36 μg/mL). These fast decreases were due to the rapid elimination of MTX from the kidney and sequestration into different organs. The clearance rate (CL), half-life (t_1/2β_), and area under the curve (AUC) of free MTX were 0.086 ± 0.03 L/h, 5.23 ± 2.72 h, and 62.42 ± 21.51 μg·h/mL. The plasma MTX concentration observed just after administration of Lipo@MTX was lower than that of free MTX. The maximal MTX concentration was detected approximately 2 h after injection of Lipo@MTX (C_max_ = 7.49 ± 1.45 μg/mL), and a decrease in drug concentration was noted during 72 h after administration (0.33 ± 0.10 μg/mL). These data suggested the sustained release feature of the lipidic nanostructure evidenced by the decreased CL (~ 0.37-fold, P < 0.05) and improved t_1/2β_ (~ 2.41-fold, P < 0.01) compared to those of free MTX-treated mice. As a result, the MRT (~ 2.79-fold, P < 0.01) and AUC_0-t_ (~ 2.57-fold, P < 0.05) of Lipo@MTX were significantly extended compared to those of free MTX-treated mice. The results conspicuously designated the long circulation properties of the PEG-decorated liposomes. Additionally, MTX encapsulation into nano-shells could protect this drug from adverse conditions, facilitating controlled MTX release to improve drug stability in blood circulation. The drug-time curve of Cy-Lipo@MTX was similar to that of Lipo@MTX. The slightly lower AUC_0-t_ of Cy-Lipo@MTX was probably due to the faster distribution of Cy-Lipo@MTX to the tumor site where ENT1 was expressed abundantly. In brief, our results suggested that PEG-modified liposomes achieved better MTX retention in circulation. The long circulation feature of the nanocarriers provided Cy-Lipo@MTX with more opportunities to anchor to ENT1.

**Table 2:** Main parameters of MTX formulations after vein injection in rats

Parameters	Free MTX	Lipo@MTX	Cy-Lipo@MTX
AUC_0-t_ (μg·h/mL)	62.42 ± 21.51	160.22 ± 47.02^*^	134.27 ± 18.19^*^
MRT_(0-t)_ (h)	6.27 ± 2.52	17.52 ± 2.47^**^	17.63 ± 0.33^**^
t_1/2β_ (h)	5.23 ± 2.72	12.60 ± 1.51^*^	13.23 ± 0.41^*^
Vss (mg/kg/(mg/mL))	0.58 ± 0.10	0.60 ± 0.26	0.70 ± 0.08
CL (L/h)	0.086 ± 0.03	0.032 ± 0.01^*^	0.037 ± 0.005^*^
C_max_ (μg/mL)	13.52 ± 1.96	7.49 ± 1.45^*^	6.79 ± 1.25^**^

^*^P < 0.05, **P < 0.01, *vs* free MTX

In vivo biodistribution studies were performed to confirm the efficiency of Cy-grafted liposome-mediated intratumoral MTX delivery and the bypass of off-target tissues (Fig. 10d, e, f). Most of the free MTX was found in the kidneys and liver at 2 h post-treatment, suggesting that these organs played a primary role in the clearance of MTX. However, at 24 h post-treatment, MTX from Cy-Lipo@MTX was detectable primarily within the tumors and to a lesser extent in other tissues. The tumor-targeting effect and the EPR effect reduced Cy-Lipo@MTX accumulation within normal tissues while encouraging selective drug entry into tumors. The Cy-anchored formulations accumulated approximately 8.30 μg/g MTX in the tumors at 24 h, whereas MTX was found at concentrations of 3.68 μg/g in the heart, 5.26 μg/g in the liver, 1.37 μg/g in the spleen, 2.05 μg/g in the lung, and 1.99 μg/g in the kidney. More MTX (13.9 μg/g) was seen in all deep tumor tissues at 8 h after treatment with Cy-Lipo@MTX, which was much higher than the drug concentration in normal tissue. The MTX levels at the tumor sites were estimated as Cy-Lipo@MTX > Lipo@MTX > free MTX at the different time points. The results in this section revealed the potential targeting, tumor-specific delivery, prolonged circulation, and enhanced bioavailability features of Cy-Lipo@MTX in tumor-bearing mice. These excellent characteristics would be finally reflected in the in vivo antitumor effects of Cy-Lipo@MTX.

### In vivo choriocarcinoma therapy

**Fig. 11:**
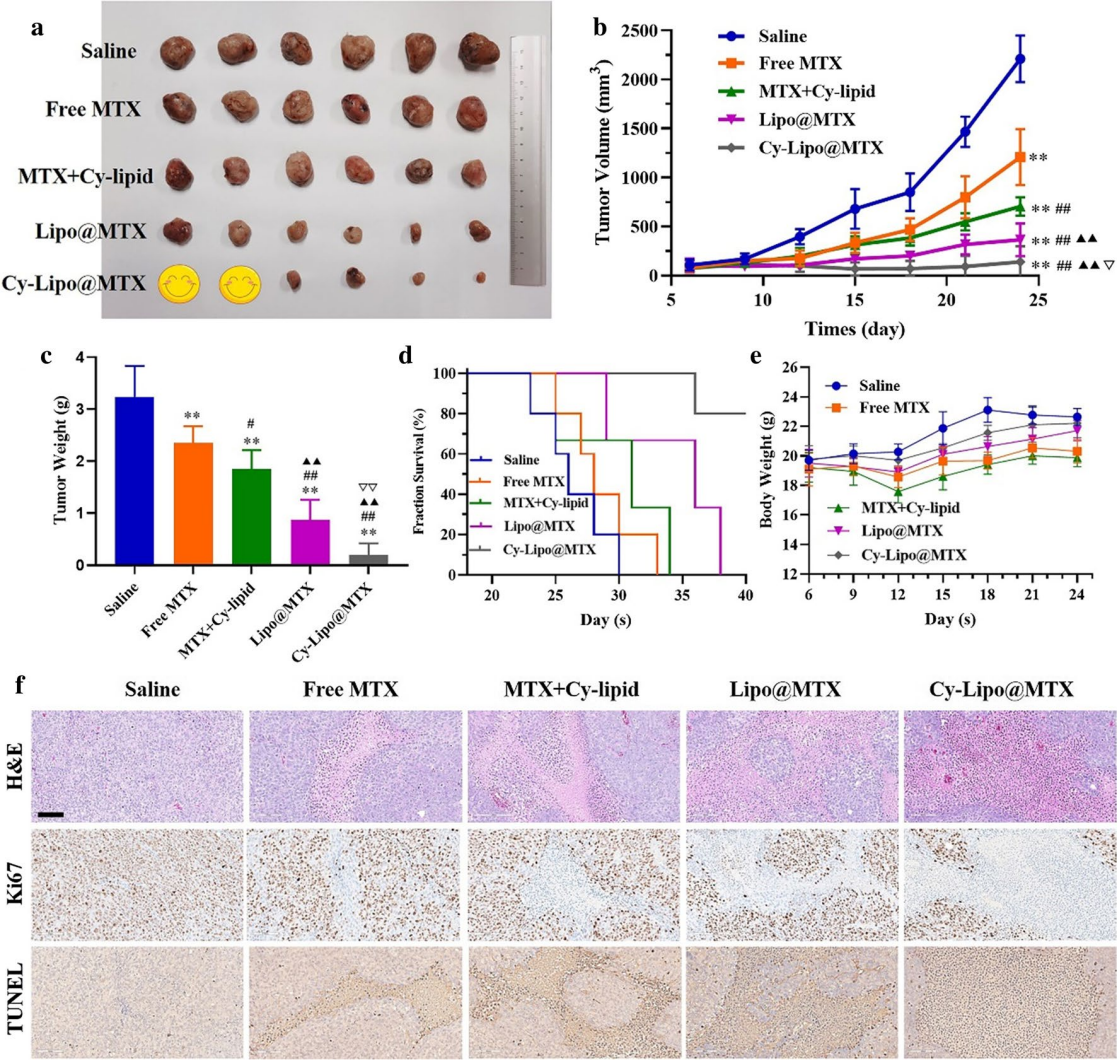
The tumor photographs on the day 24 (**a**). Tumor volume curves after intravenous injection of saline or MTX preparations (5 mg MTX-equivalent/kg body weight) from sixth day (n = 6) (**b**). Tumor weight of each group on day 24 (**c**). Kaplan–Meier survival curves of choriocarcinoma tumor-bearing mice treated with saline or MTX formulations (n = 5) (**d**). Mean body weight of the mice in different groups during the treatment (n = 6) (**e**). The H&E, Ki67, and TUNEL staining images of tumor tissues indicating the tissue necrosis, cell proliferation and cell apoptosis (**f**), scale bar is 100 µm. *P < 0.05, **P < 0.01, *vs* saline group. ^##^P < 0.01, *vs* free MTX treated group. ^▲▲^P < 0.01, *vs* MTX + Cy-lipid treated group. ^▽^P < 0.05, ^▽▽^P < 0.05, *vs* Lipo@MTX treated group

Owing to the promising in vitro and in vivo performance of Cy-Lipo@MTX, we further evaluated the antitumor activity of the designed nanodrugs against JEG-3 tumor models. When the mouse tumors grew to 50–100 mm^3^, the mice were treated with free MTX, MTX + Cy-lipid, Lipo@MTX, Cy-Lipo@MTX, or saline every three days. As shown in Fig. 11a, b, the tumor volume in the Cy-Lipo@MTX group (140.13 ± 159.40 mm^3^) was significantly reduced compared to that of the saline group (2211.35 ± 236.66 mm^3^, P < 0.01), free MTX group (1210.14 ± 285.62 mm^3^, P < 0.01), MTX + Cy-lipid group (705.42 ± 94.21 mm^3^, P < 0.01), and Lipo@MTX group (366.36 ± 166.49 mm^3^, P < 0.01) on day 24. Correspondingly, the tumor weight in the Cy-Lipo@MTX group (0.19 ± 0.22 g, P < 0.01) was significantly less than those in the saline group (3.24 ± 0.60 g, P < 0.01), free MTX group (2.35 ± 0.32 g, P < 0.01), MTX + Cy-lipid group (1.85 ± 0.36 g, P < 0.01), and Lipo@MTX group (0.87 ± 0.38 g, P < 0.05) at the end of the treatment cycle (Fig. 11c). The tumor growth inhibition index of the Cy-Lipo@MTX group was 93.66%, which was 2.06-, 1.38-, and 1.12-fold higher than that of free MTX, MTX + Cy-lipid, and Lipo@MTX groups, respectively. The tumors treated with Cy-Lipo@MTX exhibited the most potent treatment effect with almost no tumor growth after six times of treatment. In the in vitro study, the cytotoxicity of MTX + Cy-lipid was stronger than that of Lipo@MTX; however, the results were the opposite in the in vivo studies. These inconsistent results were primarily caused by three factors: the enhanced cellular uptake effect of the liposome-encapsulated MTX, the EPR effect of the nano-sized formulations, and the improved pharmacokinetic characteristics compared to the free drugs. Cy-Lipo@MTX, which combined the targeted and tumor-sensitive drug release features, exhibited optimal antitumor inhibitory activity. To evaluate the therapeutic effects of the MTX preparations on mice bearing larger tumors (approximately 300–500 mm^3^), a survival study was applied. As displayed in Fig. 11d, the median survival time of the mice treated with Cy-Lipo@MTX (45 days) was longer than that of the mice administered saline (26 days), free MTX (28 days), MTX + Cy-lipid (34 days), or Lipo@MTX injection (35 days). The H&E staining results demonstrated that Cy-Lipo@MTX exhibited the largest area of tumor tissue necrosis among the MTX formulations. Furthermore, the Ki67 and TUNEL staining results suggested that the Cy-Lipo@MTX-treated mice had the least amount of tumor cell proliferation and the most tumor cell apoptosis (Fig. 11f). These results demonstrated the promising anti-choriocarcinoma effects of Cy-Lipo@MTX.

### Safety evaluation

In the safety analysis, no loss of body weight was observed in the liposome formulation-treated groups (Cy-Lipo@MTX and Lipo@MTX). However, severe weight loss was detected in the free MTX- and MTX + Cy-lipid-treated mice (Fig. 11e). This might be due to severe free drug toxicity. At the end of treatment, no significant tissue destruction or damage was found in the H&E-stained slice images of the major organs in the Lipo@MTX- and Cy-Lipo@MTX-treated groups (Additional file 1: Fig. S6). However, there were many edematous glomeruli (indicated by the yellow arrows) in the free MTX- and MTX + Cy-lipid-treated mice, which indicated that long-term exposure to MTX could cause kidney toxicity (Additional file 1: Fig. S6). The hemolysis experiments indicated that the prepared Cy-Lipo@MTX were safe for intravenous injection (Additional file 1: Fig. S7). Serum biochemical parameters (alanine aminotransferase (ALT), aspartate aminotransferase (AST), and blood urine nitrogen (BUN)) supported the safety profile of Cy-Lipo@MTX (Additional file 1: Fig. S8). The promising biosafety profile of Cy-Lipo@MTX makes it a valuable tumor therapeutic platform, underscoring the efficacy and safety of systemic Cy-Lipo@MTX-mediated inhibition of primary human choriocarcinoma xenografts.

## Conclusion and prospective

Ligand-modified active targeting nanovehicles represent potentially viable tools that can efficiently deliver chemotherapeutic drugs to tumors. However, the high variability/heterogeneity in the expression of tumor cell receptors and immunogenicity of ligand-modified nanovehicles disrupt the efficiency of the targeting efforts due to serum proteins and other enzymes. Therefore, the development of novel tumor treatment targets is in demand. Tumor cells often overexpress nutrient transporters to ensure appropriate nutrient influx. These upregulated proteins appear to be excellent targets for active antitumor drug delivery. Compared with macromolecular ligands, the substrates of transporters are small molecules, such as nucleoside analogues, amino acids, choline, and biotin, which are nutrients with less immunogenicity and steric hindrance. The properties of the above small molecules are not easy to change during modification. In addition, transporters usually have wide substrate selectivity, such as for nutritional and drug substrates.

This research attempted to find a solution to the clinical treatment dilemma of choriocarcinoma from the perspective of transporters. ENT1, which is a membrane nucleoside transporter, was found to be highly expressed on the surface of choriocarcinoma cells, and ENT1 substrate-grafted liposomes were therefore constructed for the targeted delivery of MTX into choriocarcinoma cells. Importantly, this study proposed a drug delivery strategy using drug substrates of transporters as targeting molecules. The designed Cy-lipid, which was modified on the surface of Cy-Lipo@MTX, could not only assist with the coupling of Cy-Lipo@MTX to ENT1 but also play a synergistic antitumor role with the inclusion of MTX. Furthermore, this study elucidated that ENT1 entered the cytoplasm along with endocytic vesicles during the endocytosis process of Cy-Lipo@MTX and that the recovery of ENT1 could be attributed to endocytosed transporter recycling and de novo synthesis through overexpressed mRNA. Discovering and clarifying the function of ENT1 in the transport of nanodrugs can not only reignite the hope of chemotherapy against choriocarcinoma but also offer an encouraging way to treat trophoblast-related diseases, such as hydatidiform mole and ectopic pregnancy. In general, the transporter-guided intracellular drug delivery strategy holds great potential for choriocarcinoma therapy.

## Supplementary Information

**Additional file 1.** Additional figures and table.

## Data Availability

All data generated or analyzed during this study are included in this published article.

## Abbreviations

ENT1: Equilibrative nucleoside transporter 1
JEG-3: Choriocarcinoma cells
Cy: Cytarabine
GTN: Gestational trophoblastic neoplasia
DSPE-PEG_2__k_-Cy: Cytarabine conjugated distearoylphosphatidylethanolamine-polyethylene glycol
MTX: Methotrexate
Cy-Lipo@MTX: Cytarabine-conjugated methotrexate-loaded liposomes
Lipo@MTX: Methotrexate-loaded liposomes
Dox: Doxorubicin
CNT: Concentrative nucleoside transporters
OATs: Organic anion transporters
OCTs: Organic cation transporters
OCTNs: Carnitine/organic cation transporters
MRPs: Multidrug resistance-associated protein
P-gp: P-glycoprotein
BCRPs: Breast cancer resistance protein
DSC: Differential scanning calorimetry
MRT: Mean residence time
AUC: Area under the curve
C_max_: Peak plasma concentration
t_1/2_: Half-life
T_max_: Time to maximal plasma concentration
DL%: Drug loading rate
EE%: Entrapment efficiency
FBS: Fetal bovine serum
EPR: The enhanced permeability and retention effect
TEM: Transmission electron microscopy
ΔG: Free energy
IC_50_: The half maximal inhibitory concentration
MMP: Mitochondrial transmembrane potential
CLSM: Confocal laser scanning microscopy
HPLC: High-performance liquid chromatography
