# Supplementary material for: Nucleoside transporter-guided cytarabine-conjugated liposomes for intracellular methotrexate delivery and cooperative choriocarcinoma therapy
Source: J Nanobiotechnology. 2021 Jun 15;19:184. doi: 10.1186/s12951-021-00931-3 (PMC8207694; doi:10.1186/s12951-021-00931-3)
Supplement: Supplementary file 1 — Additional file 1. Additional figures and table. [file 12951_2021_931_MOESM1_ESM.docx]

**Supplementary Material**

**Title:** **Nucleoside Transporter-guided Cytarabine-conjugated Liposomes for Intracellular Methotrexate Delivery and Cooperative Choriocarcinoma Therapy**

**
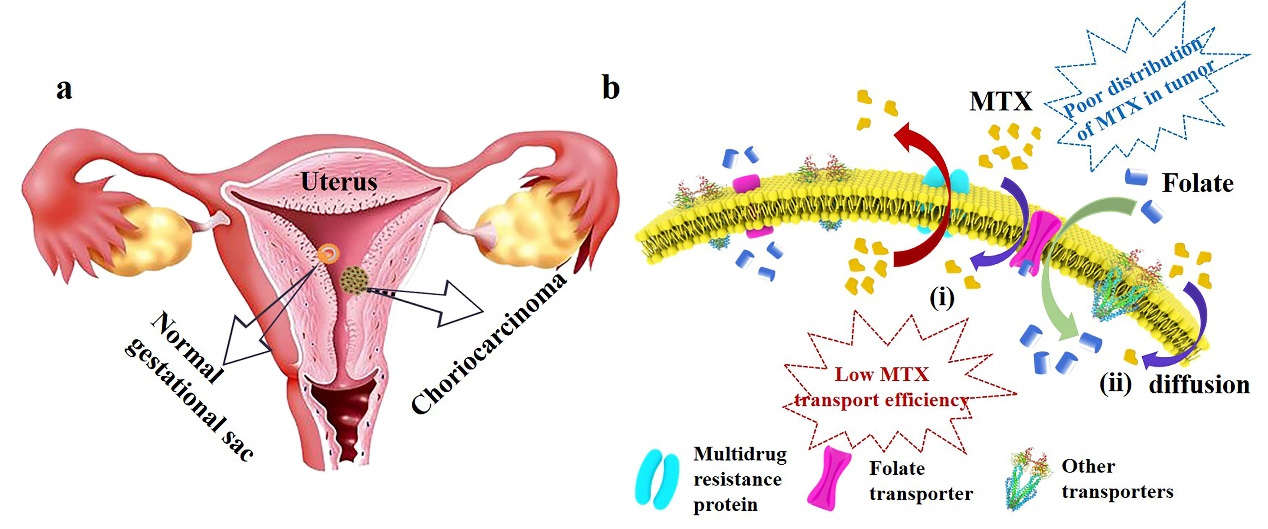
**

**Fig. S1** Schematic diagram presenting the growth of choriocarcinoma (a). Schematic diagram presenting the transport of MTX across the cell membrane through the folic acid transporter and by passive diffusion (b).


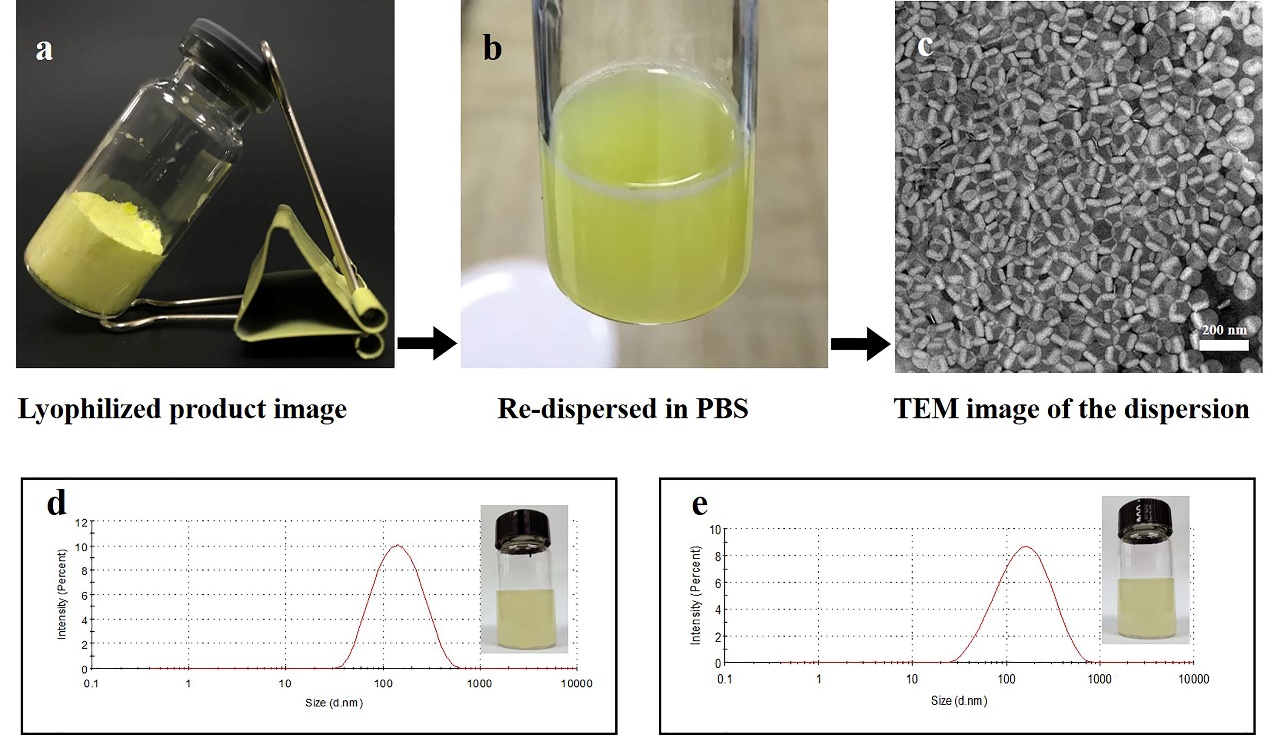


**Fig. S2** Lyophilized product image of Cy-Lipo@MTX (a). Re-dispersion of Cy-Lipo@MTX in PBS (b). TEM image of high concentration Cy-Lipo@MTX dispersion (c). The re-dispersibility (insert) and particle size distribution of lyophilized Cy-Lipo@MTX powder after storage for five (d) or nine months (e) at room temperature.

**
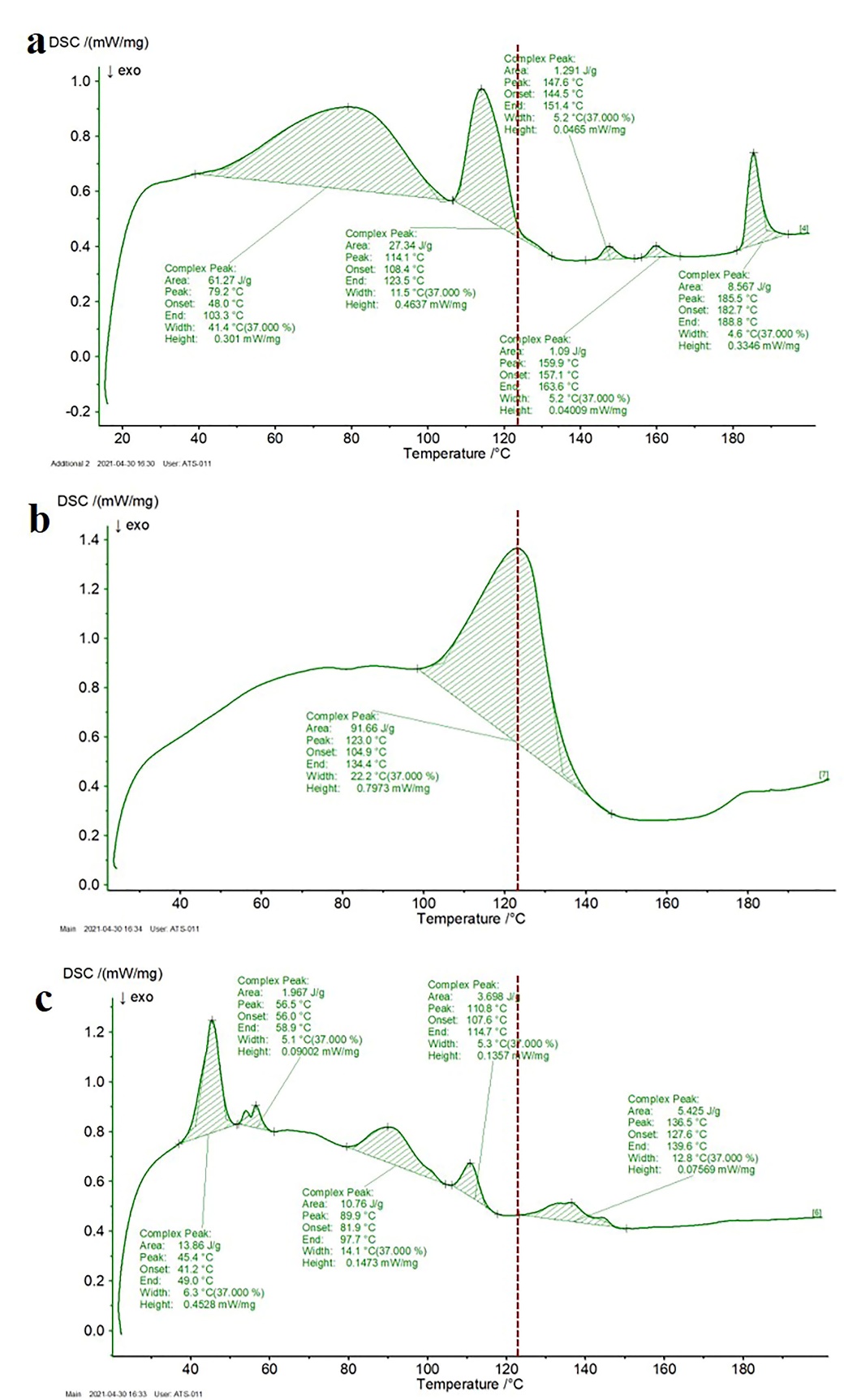
**

**Fig. S3** DSC chromatograms of physical mixture of liposome-forming material (phospholipids, cholesterol, and DSPE-PEG-Cy) (a), MTX powder (b), and lyophilized Cy-Lipo@MTX powder (c).

**
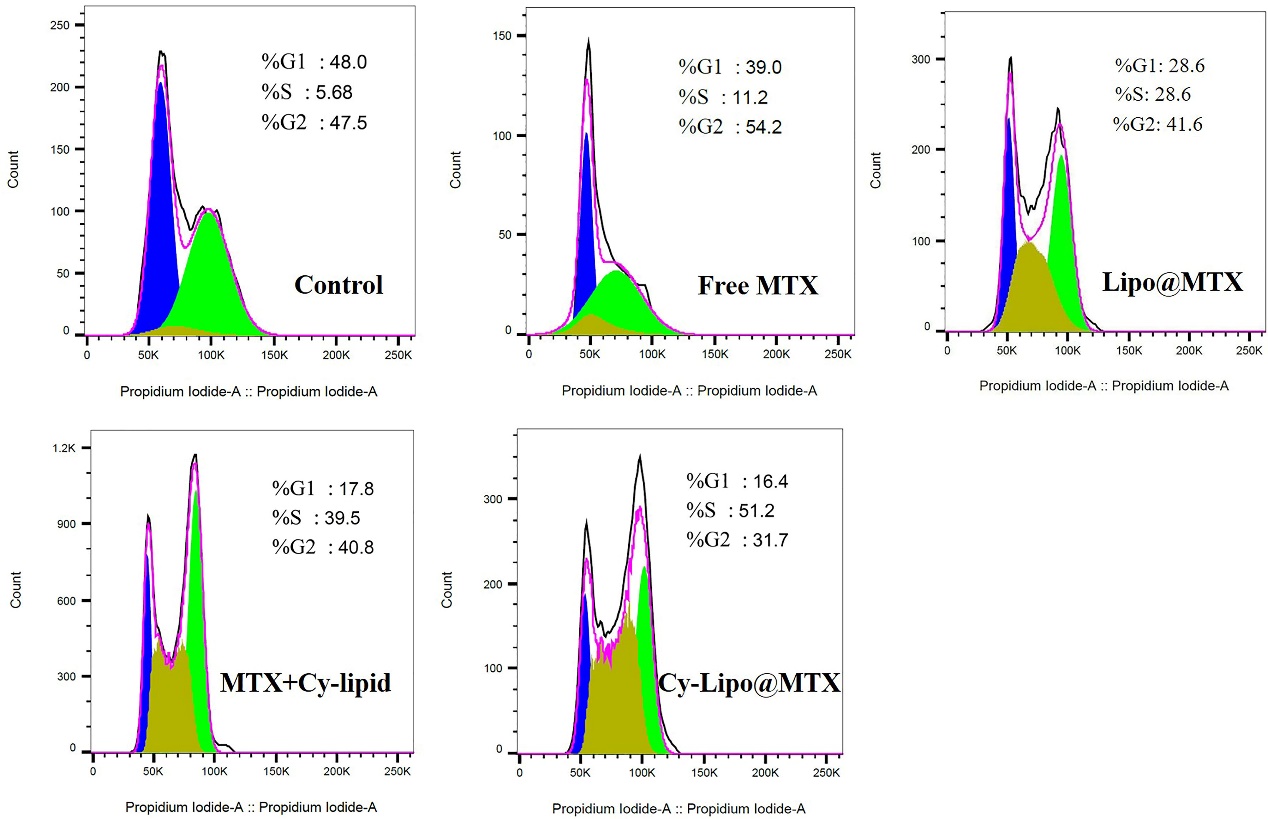
**

**Fig. S4** JEG-3 cell cycle perturbations induced by culture medium or MTX formulation-contained medium.

**
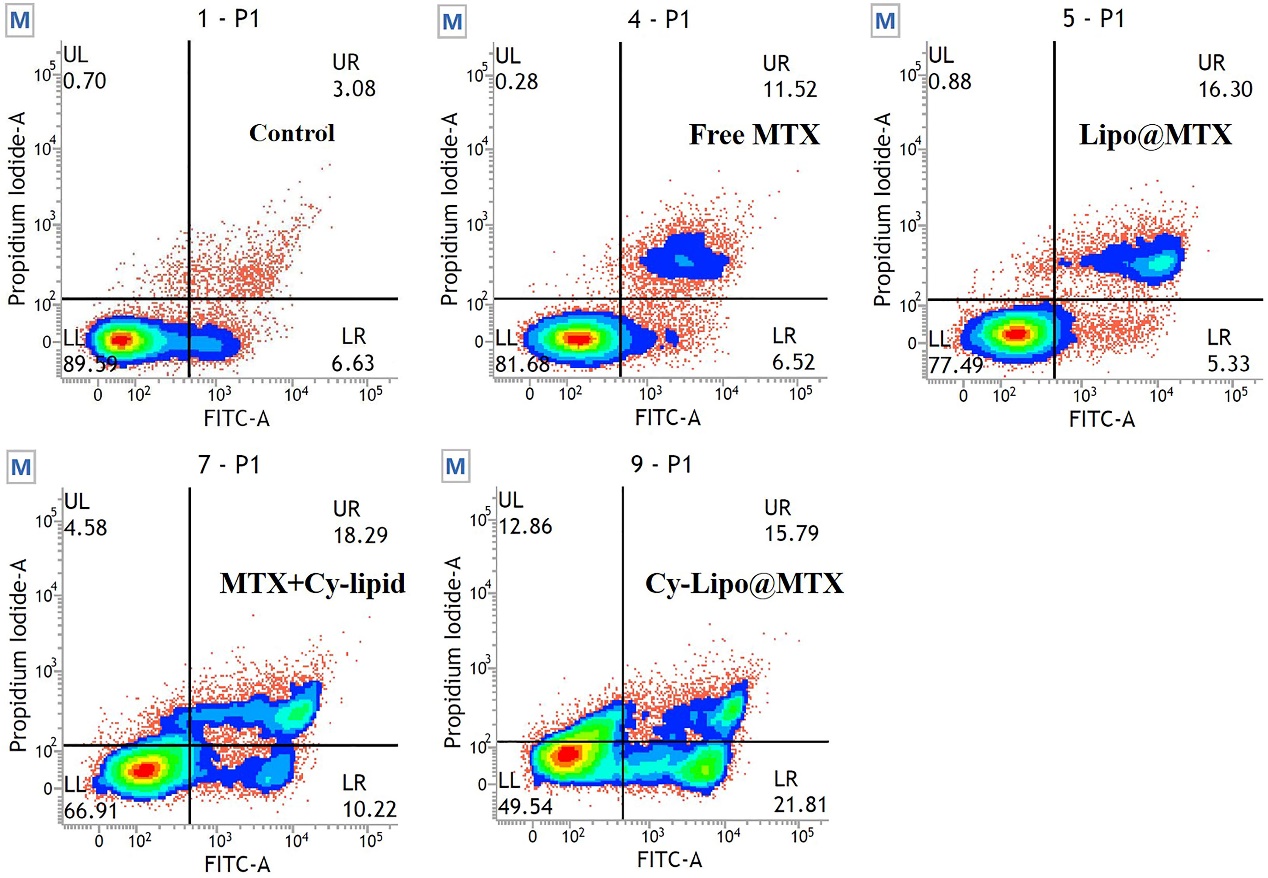
**

**Fig. S5** Analysis of apoptosis after treating JEG-3 cells with culture medium or MTX formulation-contained medium.


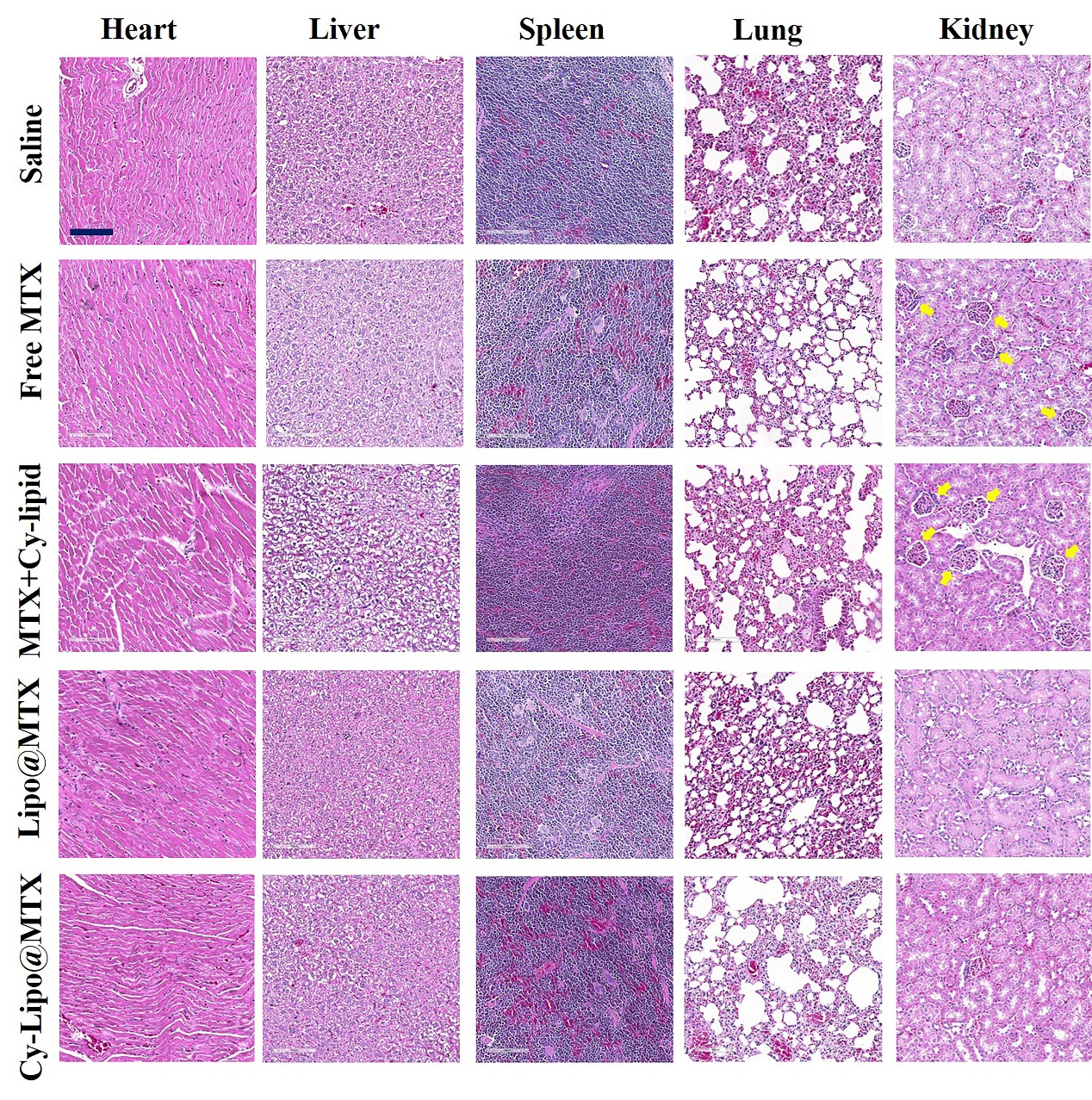


**Fig. S6** *In vivo* toxicity evaluations of various MTX formulations. H&E-stained slice images of major organs from different groups at the end of treatment, the scale bar is 100 μm. The yellow arrows represent the edematous glomeruli.

**
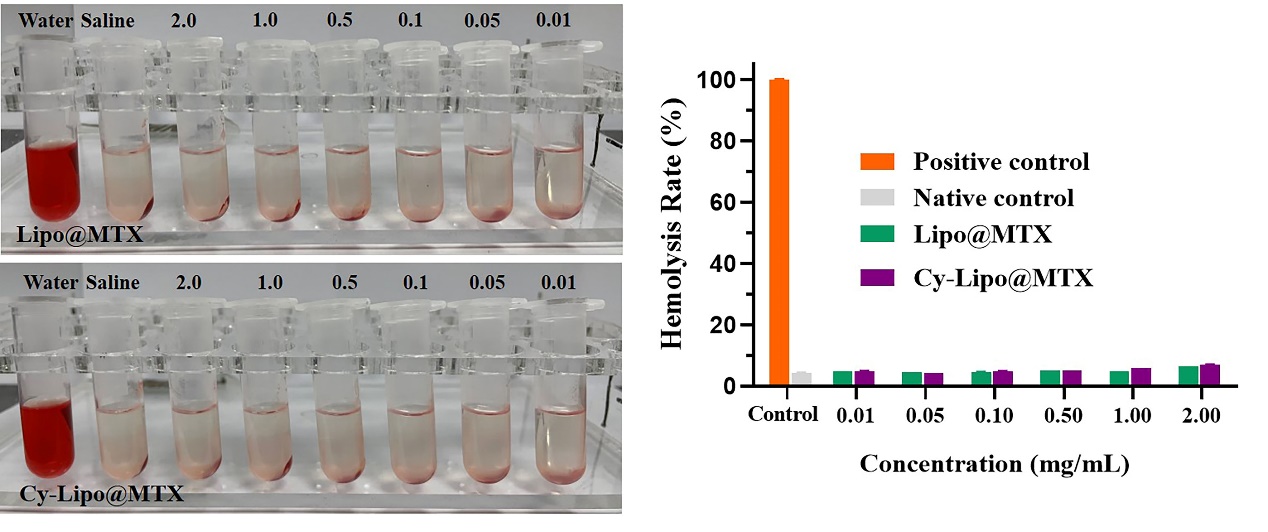
**

**Fig. S7** Hemolysis properties of different concentrations of Lipo@MTX or Cy-Lipo@MTX (0.01-2.0 mg/mL).


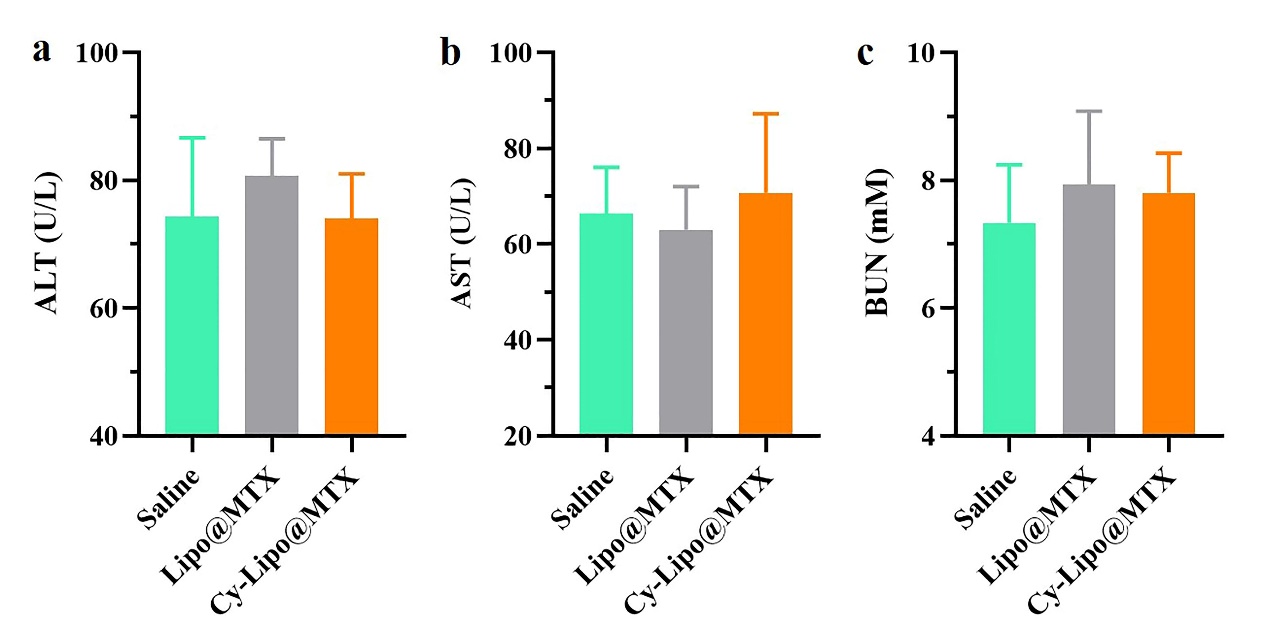


**Fig. S8** Biochemical blood analysis of saline, Lipo@MTX, and Cy-Lipo@MTX treated mice. The terms include alanine aminotransferase (ALT) (a), aspartate aminotransferase (AST) (b), and blood urine nitrogen (BUN) (c).

**Table S1** Primers list for real-time PCR

| Gene | Forward primer (5’-3’) | Reverse primer (5’-3’) |
| --- | --- | --- |
| ENT1 | CCTGGCTTTCTCTGTCTGCT; | AGTAACGTTCCCAGGTGCTG |
| ENT2 | CCCTGGATCTTGACCTGGAG; | GGTTTTCCTGGCTTCTGGG |
| CNT1 | TGAAGTGACAAGGCAAGCCA; | TCCAGCTGCTCCTGATCTCT |
| CNT2 | ACTGAGGAGCCAGAGGGAAT; | CACCGACTCCTCCTCTGGTA |
| CNT3 | GCAGGTGGCTTTCTGACAT; | AAAATGCCCCAGATGATGTGC |
| OATP1B1 | TTCAAGTGGCAATAAAAAGCCTA; | CACCCAAATGGGCTGAGTAA |
| OATP2B1 | ATACCGCTACGACAACACCA; | TGAGCAGTTGCCATTGGAG |
| P-gp | GTGGGGCAAGTCAGTTCATT; | TCTTCACCTCCAGGCTCAGT |
| BCRP | CCACTCCCACTGAGATTGAGA; | TGCGTTCCTAAATCCTACCC |
| OCTN1 | CGGAATATTGCCATAATGACC; | CAGAGCAAAGTAACCCACTGAG |
| OCTN2 | GCAGCATCCTGTCTCCCTAC; | GCTGTCAGGATGGTCAGACTT |
| OAT4 | CTGTGGAAAGTACCTCGCTCT; | CTTGAAGTCGCCCAACTCG |
| OCT3 | TCGCTCTGTTCAGGTCTGTG; | TGGATGCCAGGATACCAAAG |
| MRP1 | CATGTGGGAAAACACATCTTTG; | CAAGATCCGCGTCTTGTTC |
| MRP2 | AGTGAATGACATCTTCACGTTTG; | CTTGCAAAGGAGATCAGCAA |
| MRP3 | AGAAGGCAAAGTGCACATGA; | CCTGAAGAGTGCAGTTCTGGAT |
| MRP4 | GAAGCGCCTGGAATCTACAA; | AGAGCCCCTGGAGAGAAGAT |
| MRP5 | TTTTGCTGCAGGGCTCAT; | GGTTCCAGGGCTCACAGA |
